# Fibroblast-like synoviocytes mediate the generation of soluble PD-1 in an MMP-9-dependent manner: a novel target therapy for rheumatoid arthritis

**DOI:** 10.3389/fimmu.2025.1665078

**Published:** 2025-12-10

**Authors:** Chao Yang, Miao Ma, Kangjie Yu, Yi Wang, Yuting Zhang, Yan Shen, Jun Wang, Xiaoke Wang, Lulu Luo, Ya Zhao

**Affiliations:** 1 College of Life Sciences, Northwest University, Xi’an, China; 2 Department of Medical Microbiology and Parasitology, College of Basic Medical Sciences, Air Force Medical University, Xi’an, China; 3 Department of rheumatology and immunology, Tangdu hospital, Air Force Medical University, Xi’an, China; 4 Department of Pathology, Air Force Hospital of Eastern Theater, Nanjing, China; 5 Department of Nephrology, Xijing Hospital, Air Force Medical University, Xi’an, China

**Keywords:** rheumatoid arthritis (RA), fibroblast-like synoviocytes (FLS), soluble PD-1, MMP-9, PD-L1-MSA

## Abstract

**Introduction:**

Dysregulated T cell homeostasis leading to enhanced immune responses is a critical pathogenic mechanism in rheumatoid arthritis (RA). Programmed cell death protein 1 (PD-1) is a crucial immune checkpoint that modulates T cell activity. Soluble PD-1 (sPD-1) may contribute to the sustained activation of T cells. Investigating the mechanisms of sPD-1 production and its role in RA pathogenesis is essential for the development of alternative treatment strategies.

**Methods:**

A total of 122 patients with RA and 68 Health controls were enrolled in this study. We assessed PD-1 expression in T cells from patients with RA and measured sPD-1 levels in plasma, revealing a correlation between sPD-1 and disease activity progression in patients with RA. Furthermore, we examined the potential mechanisms underlying sPD-1 production in RA patients. Additionally, we evaluated the therapeutic effects of PD-L1-MSA, a fusion protein containing C-terminal PD-L1 and full-length MSA, in collagen-induced arthritis (CIA) mice.

**Results:**

Clinical analyses indicated that plasma sPD-1 levels positively correlated with indicators of disease activity. The generation of sPD-1 primarily resulted from matrix metalloproteinase-9 (MMP-9)-mediated shedding of surface PD-1 from activated T cells, with MMP-9 being derived from fibroblast-like synoviocytes (FLS). Additionally, therapeutic evaluation in collagen-induced arthritis (CIA) mice demonstrated that PD-L1-MSA, a fusion protein containing C-terminal PD-L1 and full-length mouse serum albumin (MSA), significantly mitigated pathological changes.

**Discussion:**

These results indicate that plasma sPD-1 levels are positively associated with RA disease activity, supporting its potential as a complementary biomarker for monitoring disease activity (especially in combination with traditional indicators like CRP/ESR), though its standalone diagnostic value requires further validation. Furthermore, targeting sPD-1, such as with PD-L1-MSA, represents a promising complementary therapeutic candidate for alleviating RA pathology.

## 1 Introduction

Rheumatoid arthritis (RA) is a common systemic autoimmune disease that affects approximately 0.5% to 1% of the global population. Its primary pathological features include chronic synovitis and hyperplasia of the synovial tissue (1, 2). A significant infiltration of immune cells into the synovial tissue promotes the transformation of fibroblast-like synoviocytes (FLS) into an aggressive, proliferative, pro-inflammatory phenotype, resulting in progressive inflammation of the synovium (3–5). The sustained activation of T cells may act as an initiating factor for lymphocytic infiltration and the subsequent destruction of the synovial tissue. Therefore, modulation of T cell activity represents a potential target for biological adjunctive therapy in RA (6–13).

The continuous activated CD4^+^ T cells, which infiltrated in synovial tissue, participate in pathological changes of synovial tissue of RA through directly promoting FLS activation (14, 15). Programmed cell death-1 (PD-1) and its ligands (PD-L1), play a crucial role in delivering inhibitory signals that modulate the equilibrium between T cell activation, tolerance, and immunopathology (16–18). In cancers, the expression of PD-L1 in tumor cells is upregulated, and T cell activity is inhibited through PD-1/PD-L1 signaling, resulting in immune escape of tumor cells (19). In RA, PD-L1 expression levels are significantly increased (18, 20), and normal PD-1/PD-L1 signaling plays an important role in modulating T cell activity and reducing synovial inflammation (21, 22). sPD-1 is a soluble PD-1 variant linked to clinical and pathological features and disease progression in various autoimmune diseases, including systemic lupus erythematosus (SLE) and immunoglobulin A nephropathy (IgAN) (23, 24). However, its specific role in RA remains unclear (25–27). Although ST et al. suggested that alternative splicing is a key mechanism in sPD-1 generation, the source and production of sPD-1 in RA require further investigation (28).

Matrix metalloproteinases (MMPs) are zinc-dependent endopeptidases that are essential for physiological functions such as wound healing, tissue remodeling, and extracellular matrix regulatio (29, 30). MMPs can be produced by a variety of tissue cells, which are essential in pathological processes in RA such as synovial tissue migration and invasion, cartilage degradation, bone erosion, and angiogenesis (31–34). Numerous studies have suggested that MMPs play a role in the shedding of cell surface molecules and contribute to disease progression. Zhang YT et al. demonstrated that MMP-2 might involve in the production of sPD-1,which may promote the fibrosis progression in IgAN (35). Abdellah Elhabazi et al. proposed that MMP-2 and MMP-9 can induce the release of CD100 from activated lymphocytes, producing a bioactive soluble form that aids CD8^+^ T cells in eliminating HBV in the liver (36). Giulia Angelini et al. found that MMP-9 upregulation is vital for CD31 domain cleavage on CD4^+^ T cells in acute coronary syndrome (ACS) patients, highlighting a potential therapeutic target for T-cell dysregulation in ACS (37).

Current treatments for RA predominantly rely on anti-cytokine agents and Janus kinase (JAK) inhibitors. While effective, these therapies primarily focus on specific cytokines or signaling pathways. However, sPD-1 modulates T cell activity via the PD-1/PD-L1 immune checkpoint pathway. Consequently, targeting sPD-1 fundamentally differs from current approaches by regulating overall T cell activity, potentially offering a broader immunomodulatory effect.

This study aimed to characterize the association between plasma sPD-1 levels and the clinical features of patients with RA, establishing sPD-1 as complementary biomarker for monitoring disease activity. Furthermore, we investigated the novel mechanism of sPD-1 production mediated by MMP-9 in RA, and explored sPD-1 as a therapeutic target. To evaluate this targeting strategy, we utilized the CIA mouse model and assessed the efficacy of the PD-L1-MSA fusion protein in mitigating synovial inflammation and tissue damage.

## 2 Materials and methods

### 2.1 Patient selection

This study was approved by the Human Research Ethics Committee of Xijing Hospital (Approval No. KY20213511-1) and was conducted in accordance with the Declaration of Helsinki. A total of 100 patients with RA and 68 healthy controls were recruited from two centers: the Department of Rheumatology and Immunology at Tangdu Hospital and the Department of Clinical Immunology at Xijing Hospital, both affiliated with the Air Force Medical University in Xi’an, China. Enrollment occurred from September 2022 to June 2024. The RA cohort consisted of 65 females and 35 males, while the healthy control group included 40 females and 28 males. All RA patients met the 2010 European Alliance of Associations for Rheumatology (EULAR) classification criteria. The exclusion criteria were as follows: (a) patients with incomplete clinical data, (b) patients diagnosed with other autoimmune diseases, and (c) Patients who received immunosuppressive agents within six months prior to enrollment.

The medication history of all patients in the 12 months prior to enrollment was traced through the electronic medical record system, and all patients were confirmed to meet the requirement of a 6-month immunosuppressive agents washout period. Among them, 28 patients had a “past medication history” (immunosuppressive agents were used 6–12 months before enrollment, and the washout period was completed), and 72 patients were in a “treatment-naive or long-term drug withdrawal state” (no use of the aforementioned immunosuppressive agents within 12 months before enrollment) (**Supplementary Table 1**).

### 2.2 Data and sample collection

Peripheral blood samples were collected from patients with RA using EDTA-anticoagulated tubes. Plasma was isolated within 2 hours post-collection by centrifugation at 3000 × g for 15 minutes at 4°C. Aliquots were immediately stored at -80°C until further analysis. All relevant clinical information regarding the eligible patients was retrospectively retrieved from their medical records. The complete clinical baseline data are presented in **Table 1**.

**Table 1:** Clinical baseline data of enrolled patients with RA.

Variables	DAS28				
	Overall	Re-RA	Lo-RA	Mo-RA	Hi-RA
	n=100	n=20	n=18	n=35	n=27
Female, n (%)	65 (65)	14 (70)	14 (78)	21 (60)	16 (59)
Age, years	58.14 ± 13.27	51.46 ± 13.02	55.17 ± 13.94	59.38 ± 12.17	59.33 ± 11.43
Disease duration, years	8.27 ± 5.34	6.17 ± 3.74	7.88 ± 4.21	9.33 ± 4.85	10.51 ± 5.07
Serostatus					
RF positive, n (%)	78 (78)	14 (70)	13 (72)	29 (83)	22 (81)
Anti-CCP positive, n (%)	81 (81)	14 (70)	12 (67)	32 (91)	23 (85)
Comorbidity					
Diabetes, n (%)	34 (34)	6(30)	6(33)	12(34)	10(37)
Cardiovascular, n (%)	26 (26)	5 (25)	5 (28)	9 (26)	7 (26)
Hypertension, n (%)	24 (24)	4 (20)	5 (28)	9 (26)	6 (22)
Laboratory index					
sPD-1, (ng/mL)	1.45 ± 0.94	1.21 ± 0.81	1.18 ± 0.88	1.56 ± 1.01	1.62 ± 0.91
MMP-9, (ng/mL)	182.8 ± 91.7	147.3 ± 87.5	151.9 ± 77.8	178.6 ± 34.1	221.3 ± 61.1
TNF-α, (pg/mL)	60.17 ± 38.84	39.14 ± 22.47	44.68 ± 25.91	58.26 ± 31.44	62.13 ± 33.62
IL-1β, (pg/mL)	26.51 ± 11.25	18.12 ± 7.55	22.65 ± 9.21	25.81 ± 9.84	28.61 ± 7.44
IL-6, (pg/mL)	55.34 ± 35.41	46.51 ± 17.49	52.88 ± 28.61	58.60 ± 27.14	56.22 ± 25.51
ESR, (mm/hr)	49.89 ± 28.33	41.35 ± 18.83	45.32 ± 17.91	48.71 ± 26.21	52.17 ± 25.58
CRP, (mg/L)	53.69 ± 21.44	44.62 ± 15.74	50.92 ± 18.85	54.21 ± 19.42	58.69 ± 17.47
RBC, 10^12^/L	4.67 ± 0.93	4.92 ± 0.85	4.51 ± 0.97	4.58 ± 0.77	4.71 ± 0.81
WBC, 10^9^/L	5.61 (2.21)	5.83 (2.92)	5.41 (2.06)	4.74 (2.53)	5.16 (2.02)
Neutrophils, 10^9^/L	3.95 (1.17)	4.28 (1.71)	3.78 (1.01)	3.85 (0.94)	3.24 (0.68)
Lymphocytes, 10^9^/L	1.74 (0.66)	1.80 (0.98)	1.70 (0.53)	1.68 (0.58)	1.65 (0.42)

Continuous variables presented as mean ± SD or median (IQR). RF positive, ≥20 IU/mL; Anti-CCP positive, ≥25 U/mL; SD, standard deviation; IQR, interquartile range; RA, rheumatoid arthritis; HC, healthy controls; DAS28, 28-joint Disease Activity Score; CDAI, Clinical Disease Activity Index; SDAI, Simplified Disease Activity Index; RBC, red blood cell; WBC, white blood cell.

This study evaluated the activity of RA based on the Disease activity score-28 (DAS28), Simplified Disease Activity Index (SDAI), and Clinical Disease Activity Index (CDAI) (**Table 2**). The formula of DAS28, SDAI and CDAI was listed as below:

**Table 2:** RA disease activity assessment criteria.

Status	DAS28	SDAI	CDAI
Re-RA	DAS28 ≤ 2.6	SDAI ≤ 3.3	CDAI ≤ 2.8
Lo-RA	2.6<DAS28 ≤ 3.2	3.3<SDAI ≤ 11	2.8<CDAI ≤ 10
Mo-RA	3.2<DAS28 ≤ 5.1	11<SDAI ≤ 26	10<CDAI ≤ 22
Hi-RA	DAS28>5.1	SDAI>26	CDAI>22

DAS28, 28-joint Disease Activity Score; CDAI, Clinical Disease Activity Index; SDAI, Simplified Disease Activity Index; Re-RA, Remission RA; Lo-RA, Low RA; Mo-RA, Moderate RA; Hi-RA, High RA.


$$\text{DAS}28=0.56\times \sqrt{\text{TJC}28}+0.28\times \sqrt{\text{SJC}28}+0.36\times \ln\left(\text{CRP}+1\right)+0.014\times \text{PGA}+0.96$$

SDAI=TJC28+SJC28+PGA+EGA+CRP

CDAI=TJC28+SJC28+PGA+EGA

TJC28: Count of 28 joint tenderness; SJC28: Count of 28 joint swollen; CRP: C-reaction protein; PGA: Patient global Assessment; EGA: Doctor’s global Assessment.

### 2.3 Flow cytometry

Peripheral blood samples were stained with the following fluorochrome-conjugated anti-human antibodies according to the manufacturer’s protocols: APC anti-human CD279 (PD-1) (BioLegend, 621608) and FITC anti-human CD3 (BioLegend, 317306). The samples were incubated with the antibody cocktail at room temperature in the dark for 30 minutes, followed by erythrocyte lysis using red blood lysate buffer. The cells were then washed three times with PBS (pH 7.4) and centrifuged at 300 × g for 5 minutes at 4°C to remove unbound antibodies. The resulting cell pellet was resuspended in 100 µL of cold PBS and immediately analyzed on a Beckman Coulter CytoFLEX flow cytometer (Beckman Coulter, Germany).

### 2.4 Enzyme-linked immunosorbent assay

#### 2.4.1 Sample collecting and storage

Use an anticoagulant tube and allow samples to separate for 2 h at room temperature before centrifugation for 15 minutes at 13,000 × g. Remove plasma aliquots and store samples at -80°C.

After centrifugation of the cell culture medium for *in vitro* culture, and the supernatant was collected and frozen at -20°C.

#### 2.4.2 Sample testing

Human plasma sPD-1 levels were determined using an ELISA kit (R&D Systems, Minneapolis, MN, USA). The human plasma MMP-2, MMP-3, and MMP-9 levels were determined using ELISA kits (Boster, China). Mouse plasma sPD-1 levels were determined using an ELISA kit (Multisciences Biotech). Mouse plasma MMP-9, IL-6, and TNF-α levels were determined using an ELISA kit (Boster, China). Quantification of sPD-1, MMP-2, MMP-3, MMP-9, IL-6, and TNF-α levels was performed according to the manufacturer’s instructions. Cell supernatant was detected by the same method and complete medium was used as a blank. Samples were measured in duplicate. The intra-assay and inter-assay coefficients of variation were below 10%.

### 2.5 Cell culture and transfection

Primary FLS cells were generously donated by Prof. Zhaohui Zheng (Department of Clinical immunization, Xijing Hospital, Air Force Medical University). Jurkat cells were cultured in RPMI-1640 medium containing 10% fetal bovine serum (FBS; Gibco, Australia), 1% penicillin-streptomycin. The primary FLS were cultured in DMEM medium containing 10% fetal bovine serum (FBS; Gibco, Australia), 1% penicillin-streptomycin. Lipofectamine 2000 was used for siRNA transfection according to the manufacturer’s instructions. The transfection medium was replaced with normal medium and the cells were incubated for 6 hours for subsequent experiments.

### 2.6 Quantitative real-time PCR

Total RNA was extracted using TRIzol reagent and subsequently reverse-transcribed into complementary DNA (cDNA) using a reverse transcription supermix (R222-01, Vazyme, China). qPCR was performed using a 2 × SYBR qPCR Master Mix (Q311-02, Vazyme, China). The primers (**Supplementary Table 2**) were synthesized by Sangon Biotechnology Co. (Shanghai, China). Relative gene expression was calculated using the 2^−ΔΔCT^ method, with glyceraldehyde 3-phosphate dehydrogenase (GAPDH) serving as the endogenous reference.

### 2.7 Western blotting

Cells were lysed using RIPA lysates containing 1% protease inhibitors according to the manufacturer’s instructions. The supernatant was collected and the protein concentration was determined using a bicinchoninic acid protein assay kit (Boster, China). Next, SDS-PAGE was performed to separate the total proteins. The proteins were then transferred to PVDF membranes and blocked with 5% skim milk for 1 hour. Afterward, the corresponding primary antibodies (anti-human MMP-9 (Proteintech, 10375-2-AP, 1:1,000), anti-human β-actin (CWbio, CW0096, 1:1,000)) were added, and the mixture was incubated overnight at 4°C. The membranes were then washed thrice with TBST. The secondary antibody was then added, incubated at room temperature for 1 hour, washed three times with TBST, and developed. Image software was used to analyze the gray value of each band and β-actin was used to normalize the gray values of the target proteins for statistical analysis.

### 2.8 Animal procedures and treatment

#### 2.8.1 Collagen-induced arthritis model

All animal procedures were approved by the Institutional Animal Care and Use Committee (IACUC) of the Air Force Military Medical University (No. IACUC-20200407). Male DBA/1J mice (6–8 weeks old, weighing 20–25 g) were housed under controlled conditions with a 12-hour light/dark cycle and had ad libitum access to food and water. Following a 7-day acclimatization period, collagen-induced arthritis (CIA) was induced via intradermal injection of 100 μg of chicken type II collagen emulsified in Complete Freund’s Adjuvant (CFA; Chondrex) on Day 0, followed by a booster injection with Incomplete Freund’s Adjuvant on Day 15. Control mice received saline injections. Starting on Day 1 post-immunization, the treatment group received intraperitoneal injections of PD-L1-MSA fusion protein (5 mg/kg body weight) every 48 hours for a total of 10 doses, while the CIA control group received equivalent volumes of saline. On Day 42, mice were placed in a transparent airtight chamber (volume: 15 L). Compressed CO_2_ (purity ≥99%) was then introduced at 7.5 L/min (50% flow rate of chamber volume per minute) for gradual displacement of ambient air, achieving unconsciousness within 2–3 minutes. After sustained respiratory arrest and loss of corneal reflex, cervical dislocation was performed as a secondary method to ensure irreversible death. All procedures complied with AVMA Guidelines (2020) and were conducted in a ventilated fume hood with operator safety protocols. Joint tissues were harvested for subsequent analysis.

#### 2.8.2 Clinical joint scores

Scoring criteria for collagen induced arthritis in mice are show in **Table 3**.

**Table 3:** Scoring criteria for collagen induced arthritis in mice.

Severity score	Degree of inflammation
0	No erythema or swelling
1	Erythema and mild swelling confined to the tarsals, ankle, or paw joint, with mild swelling at single limb
2	Erythema and mild swelling extending from the ankle to the tarsals or erythema and mild swelling of more than one toe
3	Erythema and moderate swelling extending from the ankle to the metatarsal joints or the whole paw with swelling and obvious erythema
4	Erythema and the whole paw with severe swelling encompass the ankle, foot and digits, or ankylosis of the limb, and dysfunction of the above joints

#### 2.8.3 IHC staining

The tissues underwent fixation, decalcification, dehydration through an ethanol gradient, paraffin embedding, and sectioning at a thickness of 5 μm. The sections were incubated at 60°C for 2 hours, then deparaffinized and rehydrated. Following washes with PBS, antigen retrieval was performed for 15 minutes, followed by blocking of endogenous peroxidase for 10 minutes and serum for 15 minutes. The sections were incubated overnight at 4°C with primary antibodies: Anti-MMP-9 (Proteintech, 10375-2-AP; 1:3,000), Anti-vimentin (Servicebio, GB11192; 1:200) and Anti-CD3 (Proteintech, 17617-1-AP; dilution 1:100). After additional PBS washes, the sections were incubated with a biotin-labeled secondary antibody for 20 minutes, treated with streptavidin-horseradish peroxidase (HRP), and developed using 3,3’-diaminobenzidine (DAB). Counterstaining was performed with hematoxylin, followed by dehydration and mounting with neutral gum. The stained sections were imaged using light microscopy.

#### 2.8.4 H&E staining

The tissue sections were deparaffinized, hydrated using a gradient of alcohol, stained with hematoxylin and eosin, dehydrated with a gradient of alcohol, and then sealed with neutral gum. Histopathological changes were observed and documented using an optical microscope.

#### 2.8.5 Masson staining

The paraffin sections were rehydrated and stained with hematoxylin and eosin, as well as Masson’s trichrome. Histopathological changes were observed and documented using an optical microscope.

#### 2.8.6 Safranin O staining

After rehydration, the sections were immersed in a Safranin O staining solution for 3 minutes, washed with distilled water for 1 minute, and then immersed in a fast green staining solution for 2 minutes. Following this, the sections were washed again with distilled water for 1 minute, differentiated with 1% glacial acetic acid for 1 minute, dehydrated with 95% ethanol, and finally discolored with xylene. Histopathological changes were observed and recorded using an optical microscope.

#### 2.8.7 TRAP staining

The substrate solution was prepared according to the instructions and placed in a 37°C water bath for 10 minutes. The sections were then mixed with the substrate solution and incubated for 1 hour, followed by counterstaining with haematoxylin for 2 minutes. Images were obtained after mounting with a neutral gum solution. Positive staining appeared dark red in the cytoplasm and blue-purple in the nucleus.

### 2.9 Statistical analysis

Data were processed and analyzed using GraphPad Prism software version 8.0. Quantitative data are expressed as the mean ± standard deviation. Statistical analyses were performed using Student’s t-test for comparisons between two groups and one-way analysis of variance (ANOVA) for comparisons among multiple groups. Normal tests were performed before t-tests, and the sample data followed a normal distribution. Statistical significance was set at p< 0.05.

## 3 Results

### 3.1 The proportion of PD-1^+^ T cells increased with disease activity in patients with RA

**Figure 1:**
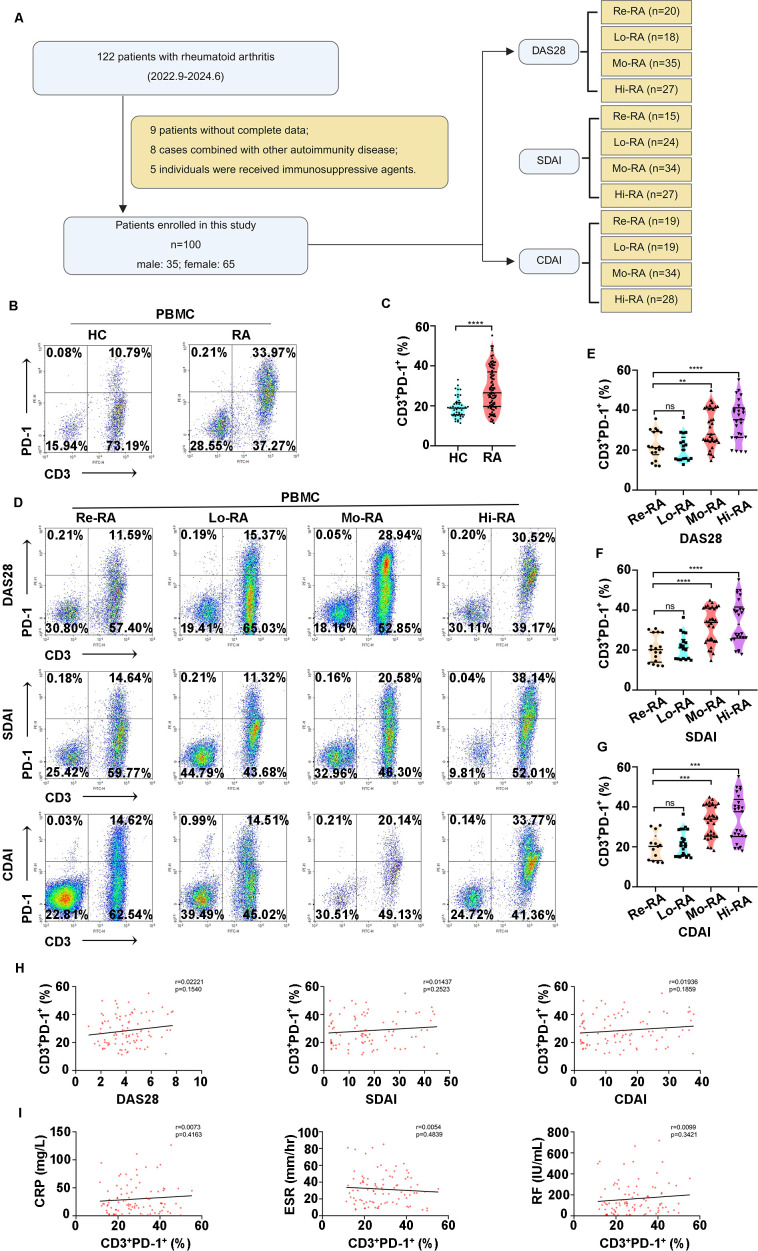
The proportion of PD-1^+^ T cells increased with disease activity indicators in patients with RA. **(A)** Study flow diagram of patient enrollment. **(B, C)** The proportion of PD-1^+^ T cells in the PBMCs of patients with RA were higher than the healthy controls (HC). **(D–G)** The proportion of PD-1^+^ T cells in PBMCs from patients in Mo-RA and Hi-RA were higher than those in Re-RA and Lo-RA. **(H)** The proportion of PD-1^+^ T cells has no correlation with disease activity score, including DAS28, SDAI and CDAI in the PBMCs of patients with RA. **(I)** The proportion of PD-1^+^ T cells has no correlation with clinical parameters, including CRP, ESR and RF in PBMCs from patients with RA. RA, Rheumatoid arthritis; HC, Health control, Re-RA, Remission RA, Lo-RA, Low RA, Mo-RA, Moderate RA, Hi-RA, High RA; DAS28, 28 joint disease activity score; SDAI, Simplified disease activity index; CDAI, Clinical disease activity index; CRP, C-reaction protein; ESR, Erythrocyte sedimentation rate; RF, Rheumatoid factor. **p<0.01, ***p<0.001, ****p<0.0001, ns, not significant.

Dysregulation of PD-1/PD-L1 signaling is a key mechanism in autoimmune diseases such as IgA nephrology (IgAN) and Systemic Lupus Erythematosus (SLE). The proportion of PD-1^+^ T cells partly indicates the degree of inflammatory progression in autoimmune diseases (16, 22, 35). Therefore, 100 RA patients and 68 healthy controls were enrolled in this study to investigate the correlation between the proportion of PD-1^+^ T cells and RA disease progression. Measures such as the 28 joint disease activity score (DAS28), Simplified disease activity index (SDAI) and Clinical disease activity index (CDAI), which characterize RA disease activity, were utilized for classification and data analysis (**Figure 1A**).

The proportion of PD-1^+^ T cells among the total T cells in PBMC was determined using flow cytometry. The proportion of PD-1^+^ T cells was significantly higher in RA patients compared to healthy controls (HC) (19.32% vs. 28.46%, p< 0.0001, **Figures 1B, C**). Additionally, according to the different disease activity assessment criteria, the proportion of PD-1^+^ T cells in Hi-RA and Mo-RA patients was notably higher than that in Lo-RA and Re-RA patients (**Figures 1D–G**). However, the correlation analysis results showed no correlation between the proportion of PD-1^+^ T cells and disease activity score or primary clinical indicators in RA (**Figures 1H, I**).

Although no direct correlation was observed between the proportion of PD-1^+^ T cells and disease activity markers or clinical indicators, PD-1 in T cells was significantly upregulated in T cells. This observation indicates that the PD-1/PD-L1 signaling pathway is a crucial component in the pathogenesis of RA.

### 3.2 Increased plasma sPD-1 were positively associated with disease activity indicators in patients with RA

The PD-1/PD-L1 pathway serves as an inhibitory signal that regulates T cell homeostasis, nonetheless, the increased expression of PD-1 in T cells exerts a small impact on the modulation of inflammatory progression in RA (16). sPD-1 is a soluble variant of PD-1, capable of combining with PD-L1; however, it lacks the ability to transmit inhibitory signals to T cells. Previous research has shown elevated serum sPD-1 levels in individuals with autoimmune diseases, correlating with various clinical markers (23, 25). Therefore, we aimed to examine the association between sPD-1 levels and clinical characteristics in patients with RA.

**Figure 2:**
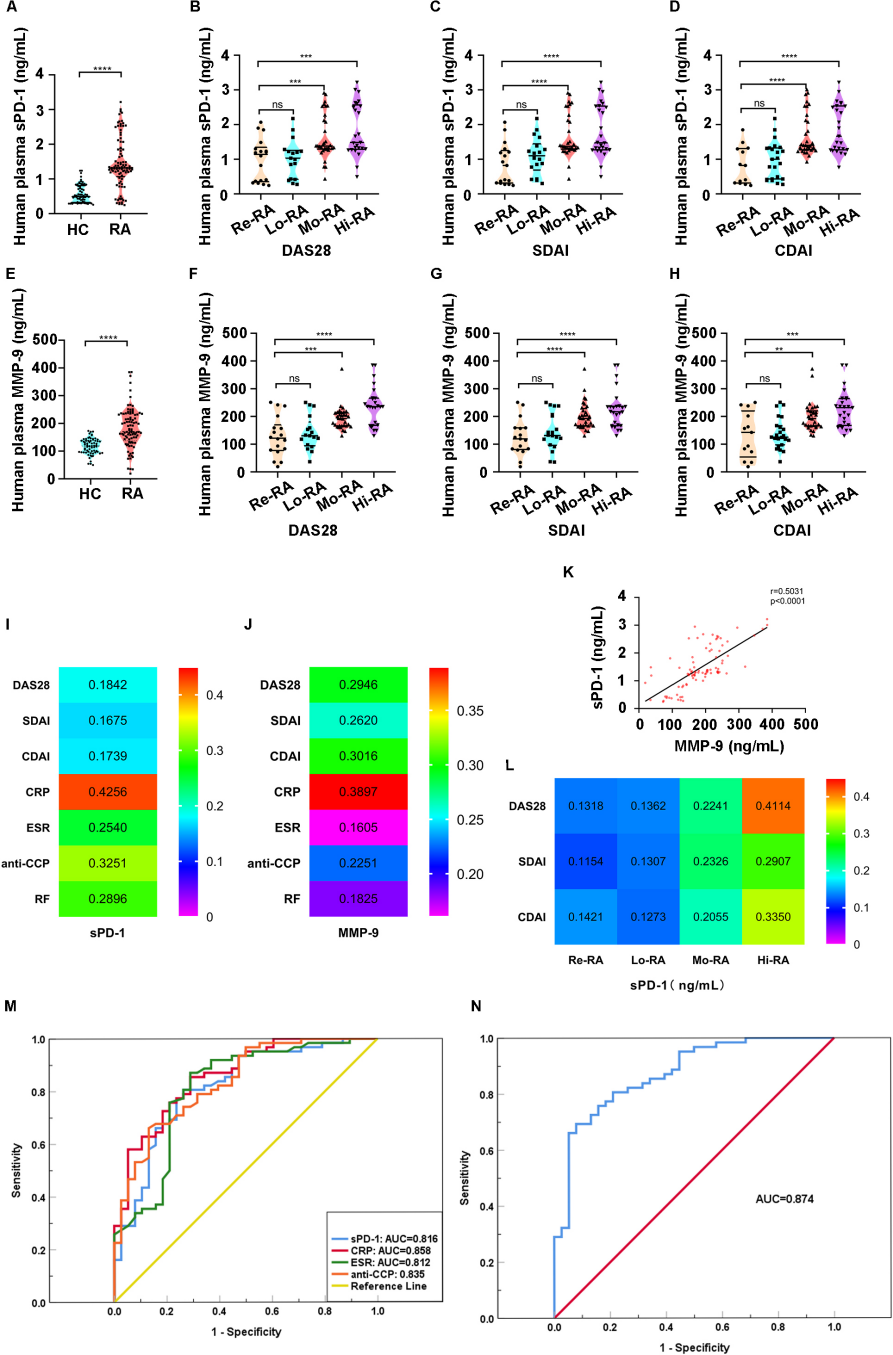
Increased plasma levels of sPD-1 and MMP-9 exhibited associations with disease activity indicators in patients with RA. **(A)** Pasma levels of sPD-1 in patients with RA were higher than Healthy controls. **(B–D)** Plasma levels of sPD-1 in Mo-RA and Hi-RA were higher than those in Re-RA and Lo-RA. **(E)** Plasma levels of MMP-9 in patients with RA were higher than HC. **(F–H)** The plasma levels of MMP-9 in Mo-RA and Hi-RA were higher than those in Re-RA and Lo-RA. **(I)** The plasma levels of sPD-1 were positively correlated with disease activity indicators (including DAS28, SDAI, and CDAI) and clinical parameter (including CRP, ESR, RF and anti-CCP) in patients with RA. **(J)** The plasma levels of MMP-9 were positively correlated with disease activity indicators (including DAS28, SDAI, and CDAI) and clinical parameter (including CRP, ESR, RF and anti-CCP) in patients with RA. **(K)** The plasma levels of MMP-9 were positively correlated with sPD-1. **(L)** Correlation analysis of plasma levels of sPD-1 with disease activity indicators and clinical parameters across different disease statuses of patients with RA. **(M)** Diagnostic efficacy comparison of sPD-1 and established biomarkers (including CRP, ESR, and anti-CCP) in patients with RA via ROC curve analysis. **(N)** Diagnostic efficacy evaluation of combined sPD-1 and CRP for patients with RA via ROC curve analysis. RA, Rheumatoid arthritis; HC, Health control, Re-RA, Remission RA, Lo-RA, Low RA, Mo-RA, Moderate RA, Hi-RA, High RA; DAS28, 28 joint disease activity score; SDAI, Simplified disease activity index; CDAI, Clinical disease activity index; CRP, C-reaction protein; ESR, Erythrocyte sedimentation rate; RF, Rheumatoid factor, Anti-CCP, anti-cyclic citrullinated peptide. ***p<0.001, ****p<0.0001, ns, not significant.

The concentration of sPD-1 in plasma was significantly higher in the RA compared than in the HC group (0.5717 ng/ml vs. 1.4529 ng/ml, p< 0.0001, **Figure 2A**). Meanwhile, Elevated sPD-1 levels were observed in the Mo-RA and Hi-RA groups compared to the Re-RA and Lo-RA groups (**Figures 2B–D**). Clinical analysis showed that sPD-1 levels were positively correlated with the DAS28 (r=0.1842, p<0.0001), SDAI (r=0.1675, p<0.0001), and CDAI (r=0.1739, p<0.0001) (**Figure 2I**). These results suggest that sPD-1 may serve as a potential biomarker for disease activity of RA. In addition, increased sPD-1 levels were positively correlated with the hematological markers C-reaction protein (CRP) (r=0.4256, p<0.0001), Erythrocyte sedimentation rate (ESR) (r=0.2540, p<0.0001), autoantibody Rheumatoid factor (RF) (r=0.2896, p<0.0001), and anti-cyclic citrullinated peptide (anti-CCP) (r=0.3251, p<0.0001) (**Figure 2I**).

Although plasma sPD-1 correlates with RA activity indices, the correlation coefficients (r ≈ 0.16–0.29) are weak. Regarding this concern, we have supplemented the analysis of the correlation between plasma sPD-1 and disease activity indicators across subgroups of RA patients stratified by different disease activity grades. The results demonstrated that, in contrast to the overall patient cohort, the correlation between sPD-1 and disease activity indicators was significantly strengthened exclusively in the high disease activity (Hi-RA) subgroup (DAS28: r=0.4110, p<0.0001; SDAI: r=0.2907, p<0.0001; CDAI: r=0.3360, p<0.0001) (**Figure 2L**). This finding indicates that sPD-1 may be more applicable for assessing the disease status of patients with high disease activity.

Using DAS28 as the grouping criterion, patients were stratified into a case group (combining Hi-RA and Mo-RA) and a control group (combining Lo-RA and Re-RA). A multivariable logistic regression model was then built to assess the association between sPD-1 and RA disease activity. The results demonstrated that after multivariate adjustment, sPD-1 exhibited an odds ratio (OR) of 2.417 (95% CI: 1.342 - 4.153) with a P-value of 0.003 (**Table 4**). These findings confirm that the correlation between sPD-1 and RA disease activity remains robust and is not confounded by the included variables.

**Table 4:** Multivariate logistic regression model in RA disease activity ^a^.

Variables	Odds ratio (95% confidence interval); P value	
	Crude	Adjusted
sPD-1 (ng/ml)	3.272 (1.451-7.241); 0.003	2.417 (1.342-4.153); 0.003

a RA disease activity was defined as MDA and HAD (DAS28>3.2).

We also compared the diagnostic efficacy of sPD-1 with that of established biomarkers in RA using ROC curve analysis. With DAS28 as the grouping standard, patients were stratified into a case group (combining Hi-RA and Mo-RA) and a control group (combining Lo-RA and Re-RA). This grouping was used to evaluate the ability of sPD-1 to discriminate between different disease activity states. The results showed that the area under the ROC curve (AUC) of sPD-1 alone was 0.816 (95% CI: 0.729 - 0.903). While this value was slightly lower than that of CRP (AUC = 0.858, 95% CI: 0.785 - 0.931; DeLong test, p = 0.08), it was significantly higher than 0.5 (p< 0.001) (**Figure 2M**). A multivariate Logistic regression model was constructed by integrating sPD-1 and CRP, and the AUC of this combined model increased to 0.874 (95% CI: 0.805 - 0.943) (**Figure 2N**).

These findings indicate elevated plasma sPD-1 levels in patients with RA, positively associated with disease activity. Thus, sPD-1 can serve as a complementary indicator to CRP, enhancing the ability to discriminate between different RA disease activity states.

### 3.3 TNF-α induces the up-regulation of MMP-9 expression in FLS from patients with RA

sPD-1 acts as a pro-inflammatory mediator in the development of specific inflammation- and immune-related diseases (12, 23, 24). Previous studies suggest that alternative splicing may be the primary molecular mechanism responsible for the generation of sPD-1 (28). Consequently, we analyzed the expression of distinct transcripts of PD-1 in PBMC from patients with RA. Agarose gel electrophoresis confirmed the presence of the PD-1 Δex3 transcript, which encodes sPD-1, in the PBMC of patients with RA (**Supplementary Figure 1A**). However, qPCR revealed that this transcript was significantly downregulated compared to that in healthy controls (**Supplementary Figure 1B**). This finding implies that traditional alternative splicing mechanisms do not sufficiently account for increased levels of sPD-1 in RA.

**Figure 3:**
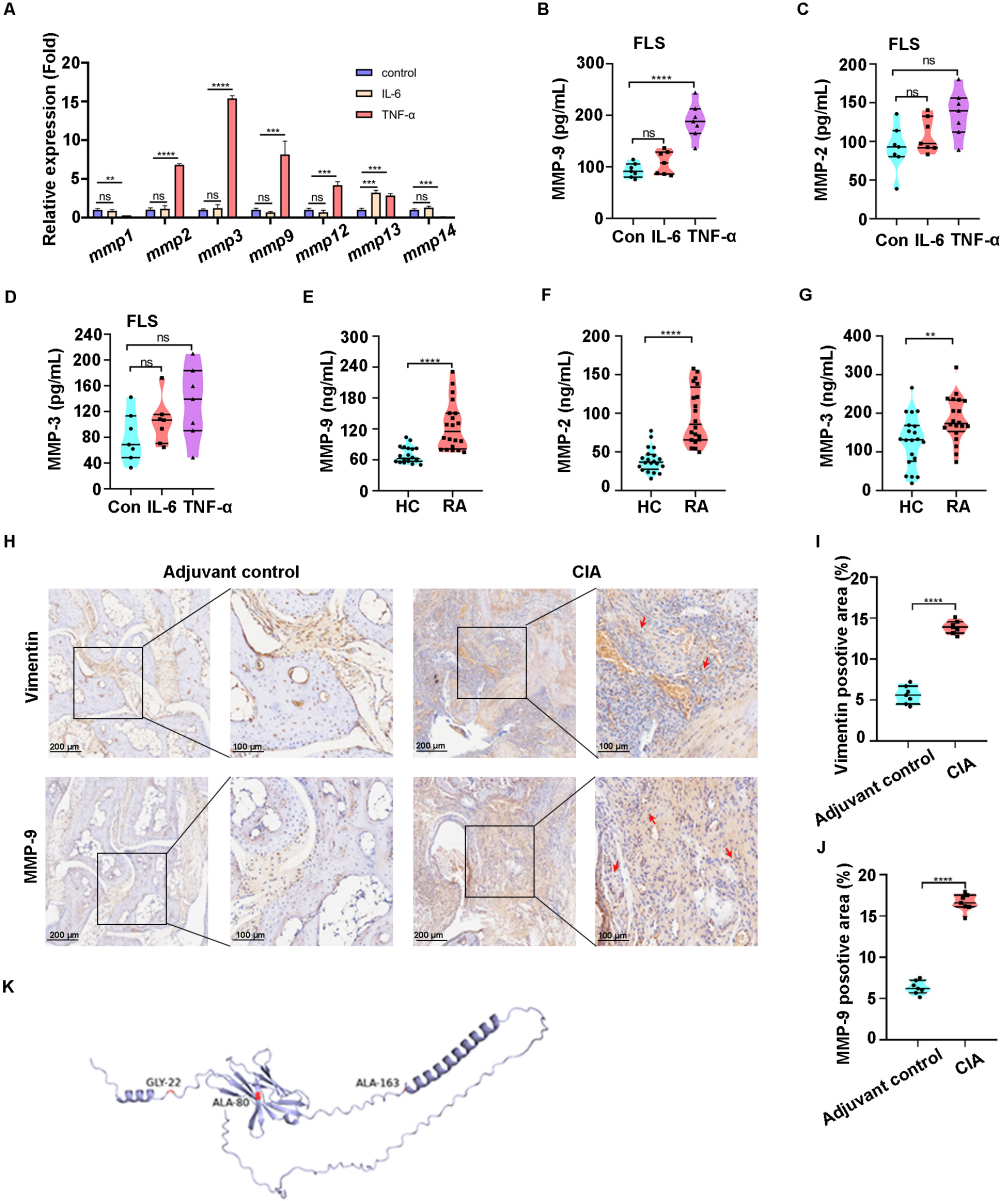
The MMP-9 expression was upregulated both in patients with RA and collagen-induced arthritis (CIA) mice. **(A)** The mRNA expression of MMP-2, MMP-3 and MMP-9 were significantly up-regulated after 20 ng/ml TNF-α treated in fibroblast-like synoviocytes (FLS). **(B–D)** The MMP-9 secretion was up-regulated after 20 ng/ml TNF-α treated in cell culture supernatant of FLS rather than MMP-2 and MMP-3. **(E–G)** The plasma levels of MMP-2, MMP-3 and MMP-9 in patients with RA are higher than HC. **(H–J)** IHC staining of vimentin and MMP-9 expression in CIA groups are higher than adjuvant controls. **(K)** Prediction of cleavage site of Human PD-1(AlphaFold ID: AF-Q15116-F1) cleaved by MMP-9. Red arrow: positive aera of IHC, **p< 0.01, ***p<0.001, ****p<0.0001, ns, not significant.

Subsequently, we investigated the potential mechanisms contributing to the generation of sPD-1 in RA. In addition to alternative splicing, hydrolysis and shedding mediated by MMPs significantly contribute to the generation of various soluble variants of cell surface molecules (29, 36, 37). FLS are the primary effector cells responsible for tissue damage in RA (1), and act as the main source of MMPs (3, 4, 9). Therefore, we stimulated FLS with TNF-α and IL-6 for 24 h to assess the expression levels of MMPs that may act as PD-1 splicing enzymes. qPCR analysis showed that the expression of MMP-2, MMP-3, and MMP-9 significantly increased in FLS following TNF-α stimulation (**Figure 3A**).

Subsequent ELISA results indicated a notable increase in MMP-9 levels in the culture supernatant of FLS after TNF-α treatment, but no significant differences were observed between MMP-2 and MMP-3 levels (**Figures 3B–D**). Subsequently, we examined the expression levels of MMP-2, MMP-3, and MMP-9 in the plasma of 20 RA patients. ELISA results indicated elevated expression of all three proteases compared to that in healthy controls, with MMP-9 showing the most significant increase (**Figures 3E–G**). Additionally, immunohistochemical analysis demonstrated a significant up-regulation of MMP-9 expression in the FLS of CIA mice (**Figures 3H–J**). In addition, by combining sequence and structural information analysis, we predicted that R-20/P-21/Gly-22, L-78/L-79/A80, and R-161/P-162/A-163 of human PD-1 may be used act as MMP-9 substrate motifs (**Figure 3K**) (38). The aforementioned results indicated that MMP-9 is significantly upregulated in RA and has the potential to cleave the extracellular domain of PD-1, thereby contributing to the formation of sPD-1.

### 3.4 The increased plasma MMP-9 were significantly associated with the indicators of disease activity in RA

To further elucidate the function of MMP-9 in the progression of RA, we detected the expression levels of MMP-9 in plasma samples from all RA patients enrolled in this study. The plasma MMP-9 was significantly higher in RA group compared to the HC (117.4 ng/ml vs 182.8 ng/ml, p<0.0001, **Figure 2E**). The levels of MMP-9 in the Mo-RA and Hi-RA groups were higher than those in the Lo-RA and Re-RA groups (**Figures 2F–H**). Clinical analysis showed that MMP-9 levels were positively correlated with the DAS 28 (r=0.2946, p<0.0001), CDAI (r=0.3016, p<0.0001), and SDAI (r=0.2620, p<0.0001) (**Figure 2J**). In addition, increased MMP-9 levels were positively correlated with CRP (r=0.3897, p<0.0001), ESR (r=0.1605, p<0.0001), ACCP (r=0.2251, p<0.0001), and RF (r=0.1825, p<0.0001) (**Figure 2J**). Consequently, a significant positive correlation was observed between plasma concentrations of MMP-9 and sPD-1 in patients with RA (r=0.5031, p<0.0001) (**Figure 2K**). Therefore, we speculated that there may be interactions between sPD-1 and MMP-9.

### 3.5 FLS-derived MMP-9 mediates the shedding of PD-1 on activated T cell surfaces to produce sPD-1

To further identify the proteases involved in sPD-1 generation, Jurkat cells were first pre-activated with monoclonal antibodies (mAbs) against CD3 and CD28 (**Supplementary Figure 2A**). Subsequently, activated Jurkat cells were stimulated with MMP-2, MMP-3, MMP-9, a disintegrin and metalloproteinase (ADAM)-10, and ADAM-17 respectively. The sPD-1 concentration in the culture supernatants and PD-1signal on activated Jurkat cells were detected by ELISA and flow cytometry. After administration of MMP-2/9, the levels of sPD-1 in the culture supernatant significantly increased in a time- and dose-dependent manner (**Supplementary Figures 2B–F**). Moreover, flow cytometry results indicated a significant decrease in the PD-1 membrane surface signal of activated Jurkat cells after 12 h of co-incubation with 20 ng/ml MMP-2/9 (**Supplementary Figures 2G, H**). The elevation of sPD-1 concentration was most pronounced in the MMP-9 treatment group.

**Figure 4:**
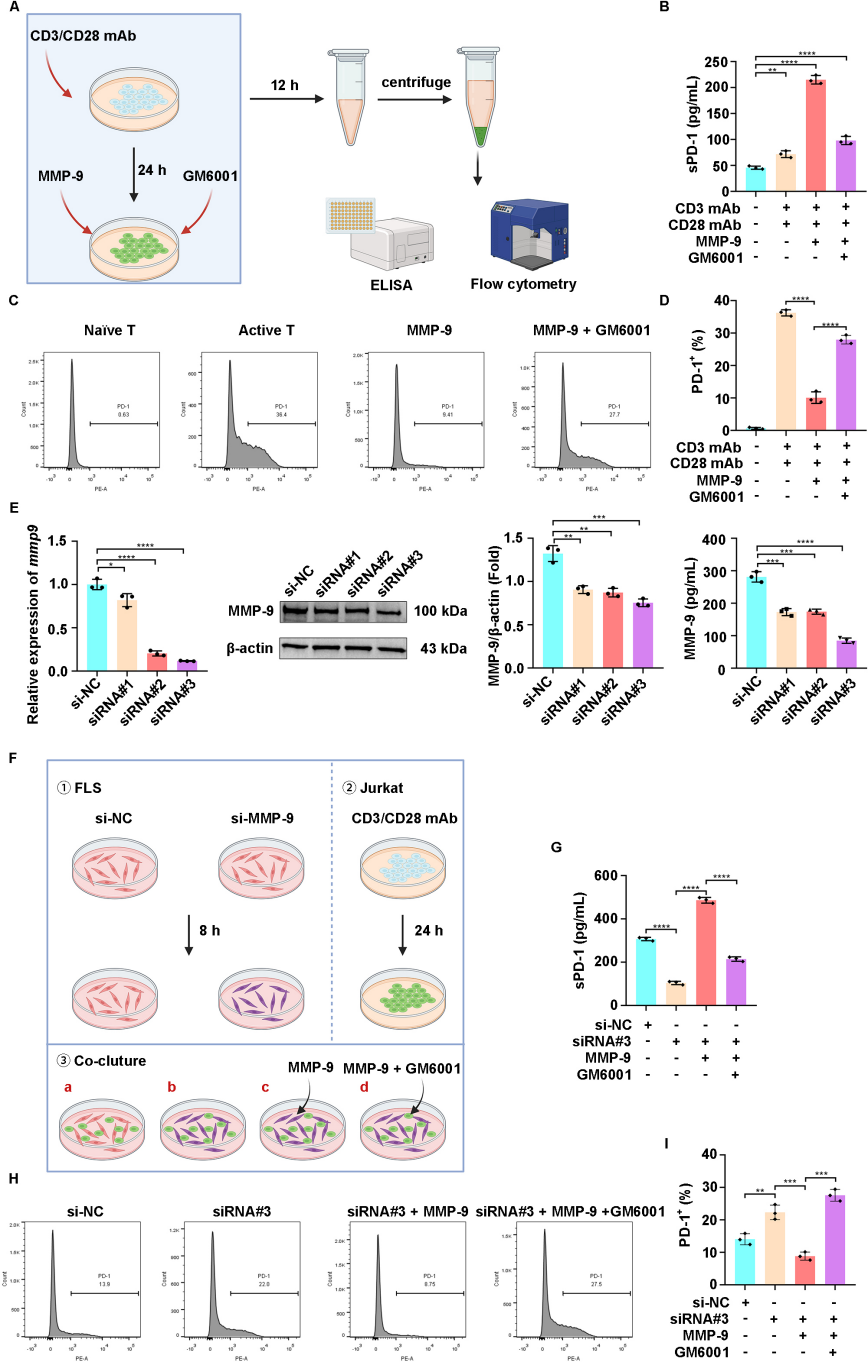
FLS-derived MMP-9 mediates the generation of sPD-1 via shedding of PD-1 on activated T cell surfaces. **(A, B)** The level of sPD-1 in the culture supernatant of Jurkat cells was significantly increased after MMP-9 stimulation, while the sPD-1 level in the GM6001 group was significantly decreased. **(C, D)** PD-1 signaling on the cell surface was significantly reduced in the MMP-9-treated group, whereas it was markedly enhanced in the GM6001 group. **(E)** The siRNA#3 knockdown the expression of MMP-9 significantly in fibroblast-like synoviocytes (FLS). **(F–I)** The knockdown of MMP-9 decreased sPD-1 levels in cell co-culture supernatants, while the PD-1 membrane surface signal in activated Jurkat cells was significantly increased. Additionally, the use of recombinant MMP-9 reversed this effect, whereas GM6001 significantly reduced sPD-1 levels in the cell culture supernatants of the recombinant MMP-9 group. *p<0.05, ** p< 0.01, ***p<0.001, ****p<0.0001.

To further verify whether MMP-9 is involved in sPD-1 production by cleaving the PD-1 on the cell surface, activated Jurkat cells were stimulated with 20 ng/mL MMP-9, with the broad-spectrum MMP inhibitor GM6001 added to abrogate MMP-9 activity (**Figure 4A**). The results demonstrated that GM6001 significantly reduced the sPD-1 level in the cell culture supernatants of the MMP-9-treated group (**Figure 4B**), while the PD-1 signal on the surface of Jurkat cells was notably upregulated (**Figures 4C, D**). MMP-9 expression was knocked down in FLS using siRNA (**Figure 4E**). Activated Jurkat cells were co-cultured with FLS in a 5:1 ratio. The co-culture results indicated that the knockdown of MMP-9 reduced the levels of sPD-1 in the supernatant, while the expression of PD-1 in Jurkat cells increased. Additionally, the use of recombinant MMP-9 reversed this effect, and GM6001 significantly reduced the sPD-1 level in the cell culture supernatants of recombinant MMP-9 group (**Figures 4F–I**). These results further suggested that sPD-1 is released from the surface of Jurkat cells via MMP-9-mediated shedding.

### 3.6 The blockage of sPD-1 mitigates the joint damage in CIA mice

**Figure 5:**
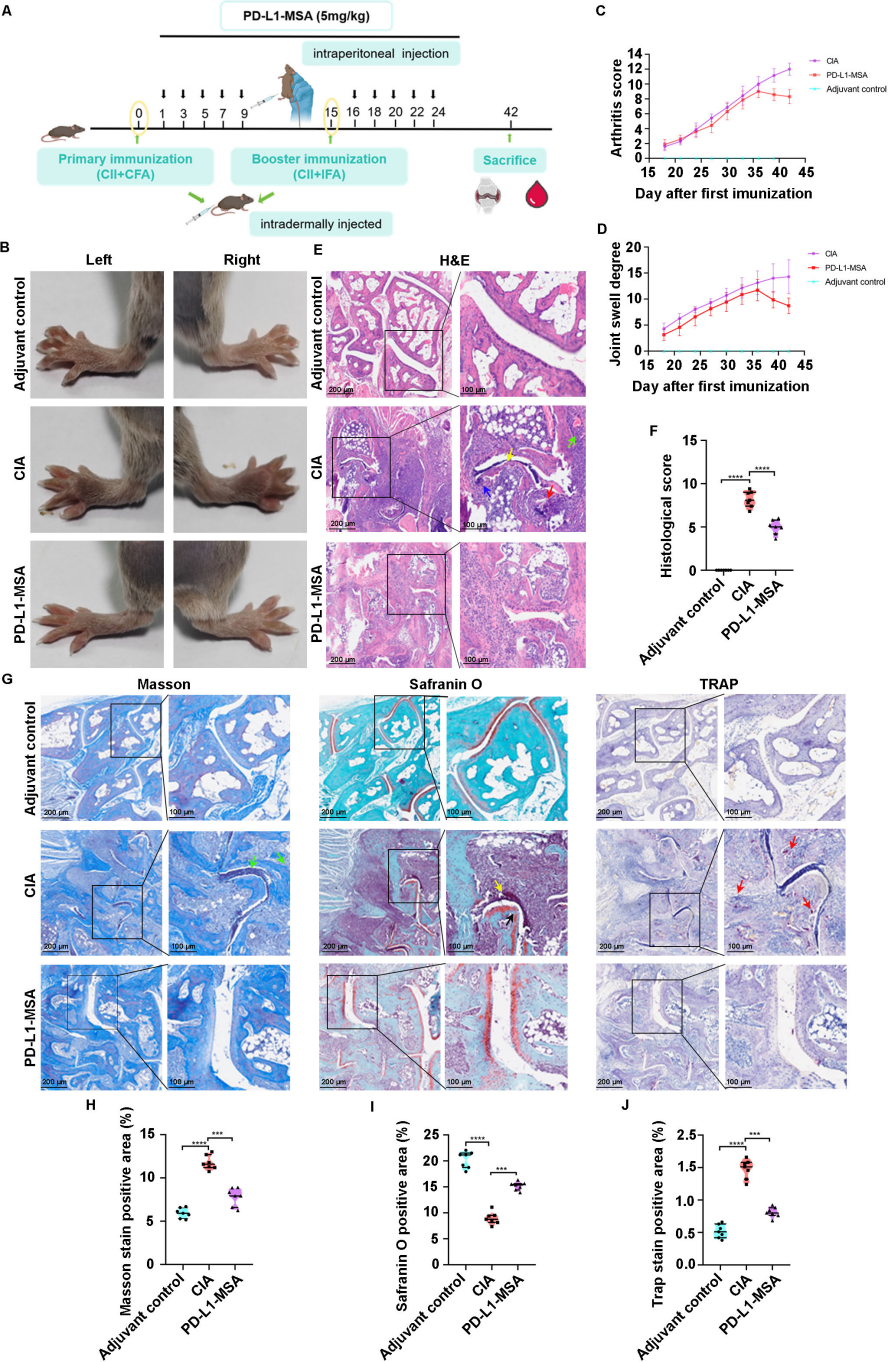
The blockage of sPD-1 mitigates the joint damage in CIA mice. **(A)** Schematic diagrams of the experimental procedure. **(B)** PD-L1-MSA significantly inhibits the joint swelling of CIA mice. **(C, D)** PD-L1-MSA significantly inhibits the index of joint swelling and arthritis index of CIA mice. **(E)** H&E staining shows that PD-L1-MSA significantly inhibits the overall pathogenic destruction in the joint of CIA mice. **(F)** Histological score for CIA mice **(G-J)** Masson and safranin O staining demonstrate that PD-L1-MSA significantly represses destruction of bone and cartilage the in the joint of CIA mice. Trap staining suggests that PD-L1-MSA inhibits osteoclast differentiation in the joint of CIA mice. Green arrow: Synovial hyperplasia; Yellow arrow: Cartilage destruction; Red arrow: Osteoclastogenesis; Blue arrow: Inflammatory cell infiltration. ***p< 0.001, ****p<0.0001.

PD-L1-MSA is a fusion protein that binds sPD-1 and inhibits its interference with the PD-1/PD-L1 pathway. PD-L1-MSA was administered to CIA mice to assess its effectiveness in reducing joint damage and inflammation (**Figure 5A**). Body weight changes in CIA mice were dynamically monitored throughout the modeling process and the administration of PD-L1-MSA. No significant differences were observed in body weight changes, as well as alanine transaminase (ALT), aspartate transaminase (AST), and blood urea nitrogen (BUN) levels of CIA mice in the PD-L1-MSA group compared with the saline control group, indicating that PD-L1-MSA has favorable safety profiles (**Supplementary Figures 3A–D**). For the detection of PD-L1-MSA immunogenicity in CIA mice, an indirect enzyme-linked immunosorbent assay (ELISA) was performed. 96-well plates were coated with 2 μg/mL PD-L1-MSA, and horseradish peroxidase (HRP)-labeled anti-mouse IgG was used as the secondary antibody. There was no significant difference in the optical density at 450 nm (OD450) values between the PD-L1-MSA group and the saline control group, demonstrating low immunogenicity of PD-L1-MSA (**Supplementary Figure 3E**).

As anticipated, swelling morphology of the paws of CIA mice was ameliorated by PD-L1-MSA treatment (**Figure 5B**). The overall therapeutic effects of PD-L1-MSA were evaluated in this study, including the degree of joint swelling (**Figure 5C**) and the arthritis index (**Figure 5D**). H&E staining revealed the alleviation of pathological changes the expression of MMP-9 was increased in CIA mice after PD-L1-MSA treatment (**Figure 5E**). Meanwhile, the histological score decreased after the PD-L1-MSA treatment (**Figure 5F**). Masson, Safranin O and TRAP staining results showed that PD-L1-MSA alleviated synovial hyperplasia, pannus formation, cartilage destruction, bone erosion, and osteoclast formation with reactive osteogenesis in the CIA model (**Figures 5G–J**). These findings indicated that the application of PD-L1-MSA as a blocker of sPD-1 successfully mitigated joint damage in the CIA model.

### 3.7 PD-L1-MSA fusion protein inhibits the hyperplasia of FLS and the infiltration of inflammatory cells in CIA mice

**Figure 6:**
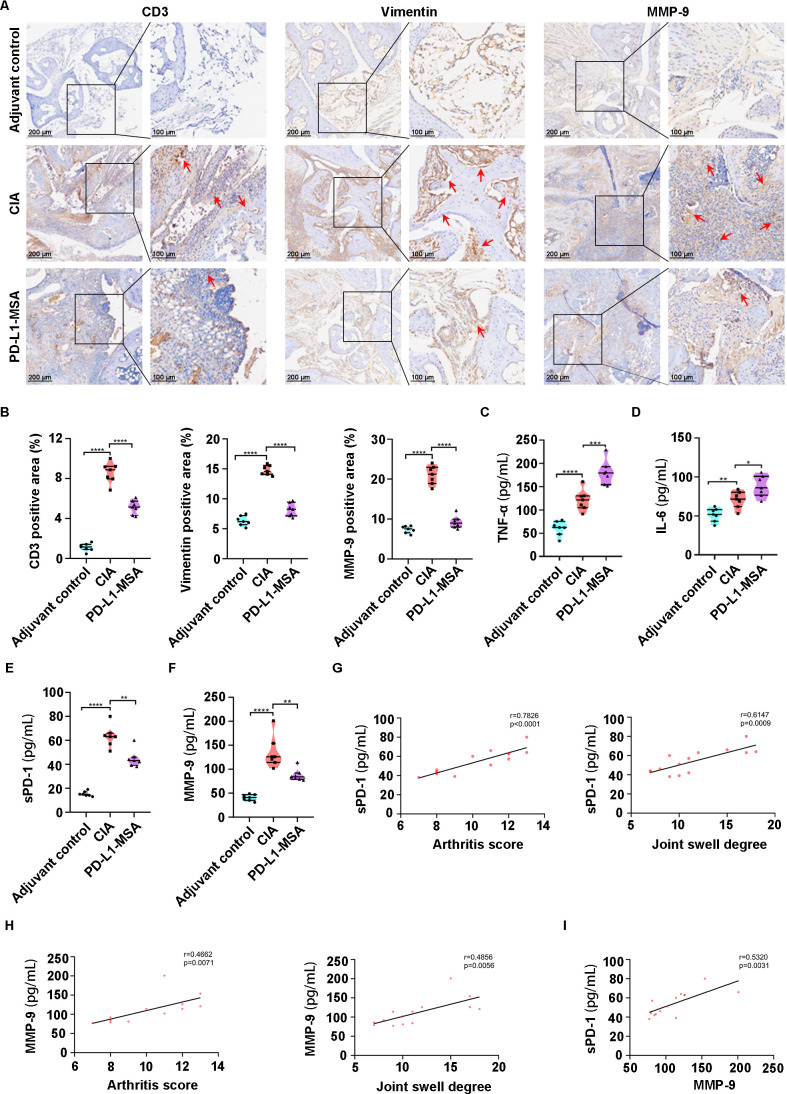
PD-L1-MSA fusion protein inhibits the FLS hyperplasia and the inflammatory cells infiltration in CIA mice. **(A, B)** IHC results of CD3 and vimentin shows that PD-L1-MSA inhibit the infiltration of CD3^+^ T cell and the hyperplasia of FLS in the CIA mice. In addition, the expression of MM9 in the joint synovium of PD-L1-MSA groups decreased. **(C, D)** The serum TNF-α and IL-6 levels were notably reduced in the PD-L1-MSA group compared to the CIA mice. **(E, F)** The serum sPD-1 and MMP-9 levels were notably reduced in the PD-L1-MSA group compared to the CIA mice. **(G, H)** The serum sPD-1 and MMP-9 levels in mice were directly associated with both the arthritis score and the extent of joint swelling. **(I)** The serum sPD-1 shows a significantly positive correlation with MMP-9. Red arrow: positive aera of IHC, *p< 0.05, **p< 0.01, ****p<0.0001.

Immunohistochemical analysis was subsequently employed to characterize the remission effect of the PD-L1-MSA fusion protein on the inflammatory progression in CIA mice. Immunohistochemical analysis of CD3 and vimentin confirmed that PD-L1-MSA represses the infiltration of T lymphocytes and hyperplasia of the FLS in the CIA joint synovium. Furthermore, compared to the CIA model group, there was a notable decrease in the expression of MMP-9 in the joint tissues of mice in the PD-L1-MSA group (**Figures 6A, B**). All of these remissions were attributed to the reestablishment of regulation of T cell activity through the blockade of sPD-1 using PD-L1-MSA. Furthermore, TNF-α and IL-6 expression levels were significantly downregulated in the PD-L1-MSA group, indicating that the PD-L1-MSA treatment effectively inhibited the systemic inflammatory response (**Figures 6C, D**). Additionally, sPD-1 and MMP-9 expression was significantly reduced in the PD-L1-MSA group compared to that in the CIA group (**Figures 6E, F**), and showed a strong correlation with the arthritis score and degree of joint swelling (**Figures 6G, H**). In addition, we observed a positive correlation between serum sPD-1 levels and MMP-9 expression in the mice (**Figure 6I**). We postulated that PD-L1-MSA treatment could effectively attenuate T cell activation by blocking the interference of sPD-1 with the PD-1/PD-L1 axis, thereby alleviating the inflammatory response and mitigating arthritis development in mice.

## 4 Discussion

This study revealed a significant increase in the proportion of PD-1^+^ T cells in the PBMC of RA patients. Although no direct correlation with disease activity markers was found, there was a notable trend linking the proportion of PD-1^+^ T cells to disease activity indicators. Increased plasma sPD-1 levels have emerged as a **complementary** biomarker for monitoring disease activity, especially in combination with traditional indicators like CRP/ESR. Our mechanistic insights suggest that sPD-1 in patients with RA likely results from PD-1 shedding on activated T cells mediated by MMP-9 derived from FLS. Furthermore, PD-L1-MSA represents a complementary therapeutic candidate for RA in the CIA mice.

The sPD-1 has been extensively studied in oncology and autoimmune diseases, demonstrating its ability to enhance T cell proliferation and activation while reducing T cell apoptosis (23). Recent research has expanded the understanding of sPD-1 beyond oncology, revealing its relevance to clinicopathological characteristics, disease stage and severity in various inflammatory and immune conditions (21, 39). Notably, sPD-1 has been identified as a pro-inflammatory factor that sustains abnormal T cell activation by disrupting PD-1/PD-L1 signaling, thereby increasing disease activity of SLE, IgAN and ankylosing spondylitis (AS) (20, 24, 35). These findings are in agreement with our findings, underscoring the pro-inflammatory properties of sPD-1. Consequently, targeting the PD-1/PD-L1 pathway through sPD-1 may effectively suppress T-cell immune responses and inhibit inflammatory reactions (40).

However, few studies have investigated the sources and mechanisms of sPD-1 production in specific pathological contexts. ST et al. identified four mRNA splice variants of PD-1 that are distinct from the full-length form (flPD-1): PD-1 Δex2, PD-1 Δex3, PD-1 Δex2, 3, and PD-1 Δex2, 3, 4. The authors suggested that sPD-1 is generated through the encoding of PD-1 Δex3, which preserves the extracellular domain of PD-1 while lacking the transmembrane domain (28). We fully acknowledge the role of alternative splicing in sPD-1 production without negating this mechanism. However, the low expression of PD-1Δex3 in RA patients may be insufficient to fully account for the increased plasma sPD-1 concentrations, which forms a critical basis for our exploration of other alternative mechanisms involved in sPD-1 production in RA. We propose that the upregulated sPD-1 expression in RA is the result of coordinated regulation by multiple distinct molecular mechanisms. Our research objective for the next phase also includes investigating the contribution of multiple mechanisms—including alternative splicing, proteolytic cleavage, and exosome-mediated pathways—to sPD-1 production.

MMPs play an important role in the pathogenesis of RA diseases (41–43). MMPs can degrade the extracellular matrix and destroy the integrity of the synovial membrane, cartilage, and bone tissue (29–31). As processing enzymes, MMPs have the ability to selectively cleave many non-matrix components present in the extracellular environment, such as cell surface receptors, cytokines, chemokines, cell-cell adhesion molecules, coagulation factors, and binding proteins, thereby participating in inflammation and immune response in RA (31, 44). The release of soluble variants from cell surface proteins seems to be a common phenomenon under both physiological and pathological conditions (31). Furthermore, ADAMs are a class of hydrolases that can cleave membrane surface molecules. Specifically, ADAM10 and ADAM17 are capable of cleaving the surface interleukin-6 receptor (IL-6R) on microglia, resulting in the production of soluble IL-6R (sIL-6R). Additionally, matrix metalloproteinase-2 (MMP-2) can cleave the surface leptin receptor (ObR) on hypothalamic cells, thereby mediating leptin resistance (45).

FLS are the predominant cellular constituents of inflamed synovial tissue and are the primary effector cells responsible for cartilage degradation and bone erosion (4). Recent research has highlighted the active role of FLS in perpetuating RA inflammation by facilitating T cell activation and promoting Th 17 differentiation. FLS is a promising therapeutic target in RA (3). FLS cells are capable of expressing all MMPs except MMP-8 and MMP-20, serving as the primary cellular source of MMPs in RA (33, 46). In our study, FLS was identified as the primary cell type, and MMP-9 was selected to elucidate the mechanism underlying sPD-1 production during RA via the proteolytic pathway following stimulation with TNF-α.

The persistent activation of T cells is a common pathogenic mechanism underlying various autoimmune diseases. To date, we have utilized PD-L1-MSA in the treatment of autoimmune disease models, including RA and IgA nephropathy. Moving forward, we also plan to apply this approach to SLE to investigate the therapeutic potential of PD-L1-MSA across a broader spectrum of autoimmune diseases. However, in the clinical management of RA, patients usually present to clinics with symptoms such as joint pain and swelling, at which stage the disease has already progressed to the active phase. Therefore, the therapeutic administration strategy is more consistent with real-world clinical needs and constitutes a critical direction for improving the translatability of PD-L1-MSA. Theoretically, if PD-L1-MSA is administered following symptom emergence, it could alleviate established joint damage by blocking sPD-1 and inhibiting inflammatory progression. Furthermore, PD-L1-MSA could bind cell-surface PD-1 as well as sPD-1. In order to compared the binding affinities of PD-L1-MSA and membrane-bound PD-1 (mPD-1), we predicted the structures of the PD-L1-MSA/sPD-1 and PD-L1-MSA/membrane-bound PD-1 (mPD-1) complexes using AlphaFold Multimer based on the amino acid sequence of PD-L1-MSA obtained in this study, respectively. The binding free energy of these complexes was calculated via the Prodigy web-based tool. The results demonstrated that PD-L1-MSA shows a higher binding affinity for sPD-1 (**Supplementary Figures 4A–C**). Therefore, in our study, we propose that PD-L1-MSA primarily exerts its effects by blocking the pro-inflammatory effects of sPD-1. Li K et al. have suggested that PD-1/PD-L1 signaling not only relies on ligand-receptor binding but may also depend on external mechanical support.

Our research has certain limitations. The current study lacks external validation and longitudinal findings in a multicenter cohort, which may limit the generalizability of sPD-1 as a complementary biomarker. In investigating the mechanism of sPD-1 production, we utilized the Jurkat T cell line. And validating the PD-1 shedding in primary RA CD4^+^ T cells would indeed significantly support disease relevance and physiological relevance. However, due to the significant heterogeneity of clinically collected T cells, we failed to complete this work. Furthermore, in the CIA model, PD-L1-MSA, as an exogenous protein, may be susceptible to hydrolysis by proteases present in mouse plasma. In future clinical practice, it will be essential to select the appropriate treatment strategy based on individual host differences.

Considering the constraints of the current study, we contend that several avenues for further research remain. Initially, although plasma sPD-1 correlates with RA activity indices, the correlation coefficients (r ≈ 0.16–0.29) are weak. These correlations achieve significance largely due to sample size, not biological strength. Thus, expanding the sample size by incorporating a larger cohort of patients would enable a more comprehensive investigation of the potential influence of sPD-1 on disease activity in individuals with RA. Furthermore, the dynamic changes of sPD-1 in patients with RA can be monitored in the future, and its clinical significance can be assessed through further cohort studies. Additionally, there is a proposal to enhance the persistence duration of PD-L-MSAs *in vivo* through affinity modification.

## 5 Conclusion

**Figure 7:**
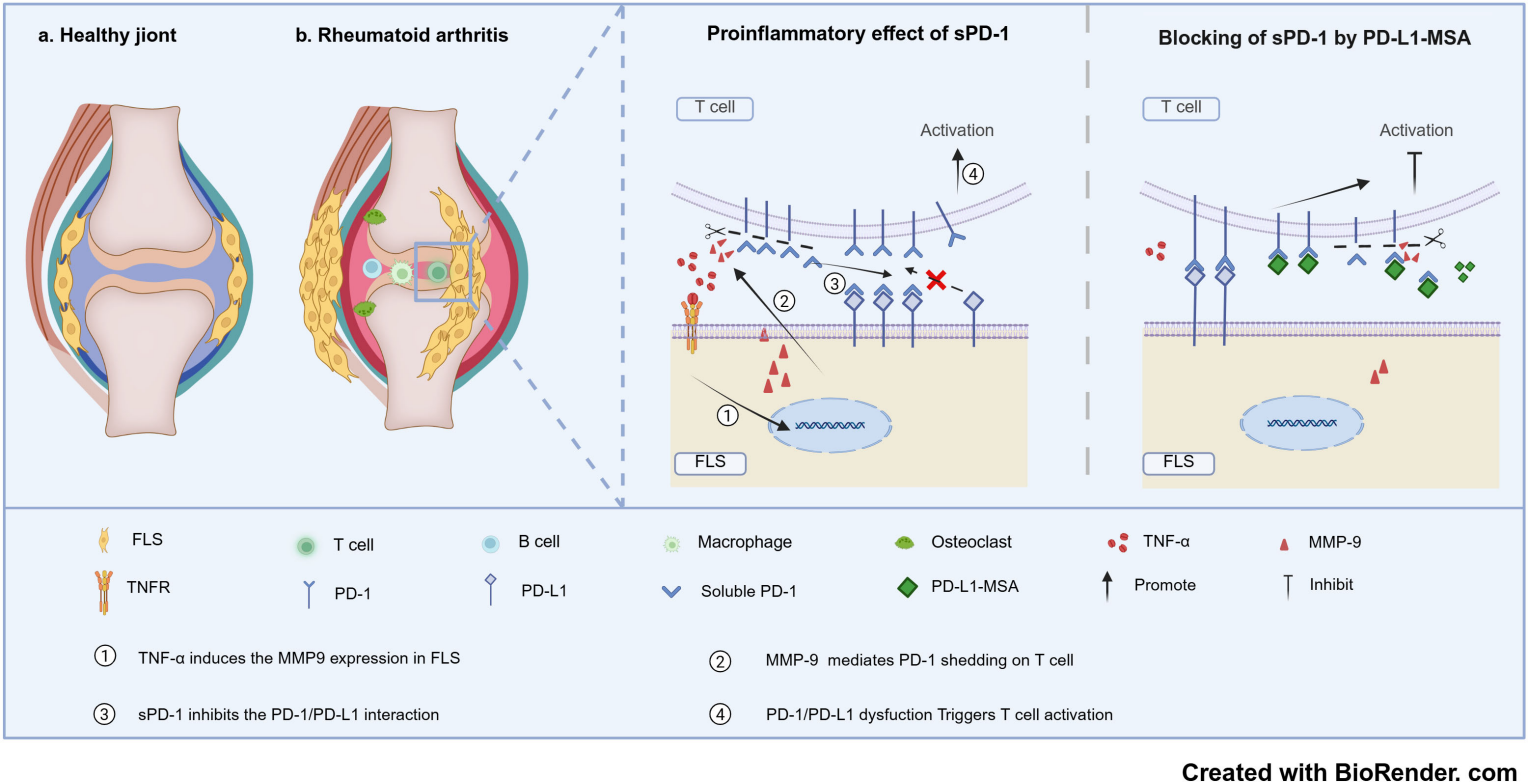
The schematic summary of the PD-1/PD-L1/MMP-9 axis in the pathogenesis of RA.

In summary, our study identified plasma sPD-1 levels as a valid biomarker for disease activity in RA and a key target for the evaluation of treatment efficacy in RA. Furthermore, we propose a novel mechanism for sPD-1 generation via FLS-derived MMP-9-dependent shedding (**Figure 7**). Finally, we used the sPD-1 blocking agent PD-L1-MSA to block sPD-1 interference in the PD-1/PD-L1 signaling pathway, achieving significant therapeutic effects in CIA mice.

## Data Availability

The original contributions presented in the study are included in the article/**Supplementary Material**. Further inquiries can be directed to the corresponding author/s.

## References

[1] Di Matteo A Bathon JM Emery P . Rheumatoid arthritis. Lancet. (2023) 402:2019–33. doi: 10.1016/S0140-6736(23)01525-8, PMID: 38240831

[2] Sparks JA . Rheumatoid arthritis. Ann Intern Med. (2019) 170:ITC1–ITC16. doi: 10.7326/AITC201901010, PMID: 30596879

[3] Bustamante MF Garcia-Carbonell R Whisenant KD Guma M . Fibroblast-like synoviocyte metabolism in the pathogenesis of rheumatoid arthritis. Arthritis Res Ther. (2017) 19:110. doi: 10.1186/s13075-017-1303-3, PMID: 28569176 PMC5452638

[4] Croft AP Campos J Jansen K Turner JD Marshall J Attar M . Distinct fibroblast subsets drive inflammation and damage in arthritis. Nature. (2019) 570:246–51. doi: 10.1038/s41586-019-1263-7, PMID: 31142839 PMC6690841

[5] Bottini N Firestein GS . Duality of fibroblast-like synoviocytes in RA: passive responders and imprinted aggressors. Nat Rev Rheumatol. (2013) 9:24–33. doi: 10.1038/nrrheum.2012.190, PMID: 23147896 PMC3970924

[6] Brown P Pratt AG Hyrich KL . Therapeutic advances in rheumatoid arthritis. BMJ. (2024) 384:e070856. doi: 10.1136/bmj-2022-070856, PMID: 38233032

[7] Lee DSW Rojas OL Gommerman JL . B cell depletion therapies in autoimmune disease: advances and mechanistic insights. Nat Rev Drug Discov. (2021) 20:179–99. doi: 10.1038/s41573-020-00092-2, PMID: 33324003 PMC7737718

[8] Mueller A-L Payandeh Z Mohammadkhani N Mubarak SMH Zakeri A Alagheband Bahrami A . Recent advances in understanding the pathogenesis of rheumatoid arthritis: new treatment strategies. Cells. (2021) 10:3017. doi: 10.3390/cells10113017, PMID: 34831240 PMC8616543

[9] Alivernini S Firestein GS McInnes IB . The pathogenesis of rheumatoid arthritis. Immunity. (2022) 55:2255–70. doi: 10.1016/j.immuni.2022.11.009, PMID: 36516818

[10] Zhao Z Hua Z Luo X Li Y Yu L Li M . Application and pharmacological mechanism of methotrexate in rheumatoid arthritis. BioMed Pharmacother. (2022) 150:113074. doi: 10.1016/j.biopha.2022.113074, PMID: 35658215

[11] Negi S Tandel N Sharma P Kumar R Tyagi RK . Aceclofenac and methotrexate combination therapy could influence Th1/Th17 axis to modulate rheumatoid-arthritis-induced inflammation. Drug Discov Today. (2023) 28:103671. doi: 10.1016/j.drudis.2023.103671, PMID: 37330038

[12] Elemam NM Hannawi S Maghazachi AA . Role of chemokines and chemokine receptors in rheumatoid arthritis. Immunotargets Ther. (2020) 9:43–56. doi: 10.2147/ITT.S243636, PMID: 32211348 PMC7074856

[13] Kerschbaumer A Sepriano A Bergstra SA Smolen JS van der Heijde D Caporali R . Efficacy of synthetic and biological DMARDs: a systematic literature review informing the 2022 update of the EULAR recommendations for the management of rheumatoid arthritis. Ann Rheum Dis. (2023) 82:95–106. doi: 10.1136/ard-2022-223365, PMID: 36368906

[14] Gravallese EM Firestein GS . Rheumatoid arthritis - common origins, divergent mechanisms. N Engl J Med. (2023) 388:529–42. doi: 10.1056/NEJMra2103726, PMID: 36780677

[15] Nygaard G Firestein GS . Restoring synovial homeostasis in rheumatoid arthritis by targeting fibroblast-like synoviocytes. Nat Rev Rheumatol. (2020) 16:316–33. doi: 10.1038/s41584-020-0413-5, PMID: 32393826 PMC7987137

[16] Keir ME Butte MJ Freeman GJ Sharpe AH . PD-1 and its ligands in tolerance and immunity. Annu Rev Immunol. (2008) 26:677–704. doi: 10.1146/annurev.immunol.26.021607.090331, PMID: 18173375 PMC10637733

[17] Zhang X Schwartz J-CD Guo X Bhatia S Cao E Lorenz M . Structural and functional analysis of the costimulatory receptor programmed death-1. Immunity. (2004) 20:337–47. doi: 10.1016/s1074-7613(04)00051-2, PMID: 15030777

[18] Sun C Mezzadra R Schumacher TN . Regulation and function of the PD-L1 checkpoint. Immunity. (2018) 48:434–52. doi: 10.1016/j.immuni.2018.03.014, PMID: 29562194 PMC7116507

[19] Barber DL Wherry EJ Masopust D Zhu B Allison JP Sharpe AH . Restoring function in exhausted CD8 T cells during chronic viral infection. Nature. (2006) 439:682–7. doi: 10.1038/nature04444, PMID: 16382236

[20] Brown JA Dorfman DM Ma F-R Sullivan EL Munoz O Wood CR . Blockade of programmed death-1 ligands on dendritic cells enhances T cell activation and cytokine production. J Immunol. (2003) 170:1257–66. doi: 10.4049/jimmunol.170.3.1257, PMID: 12538684

[21] Yi M Zheng X Niu M Zhu S Ge H Wu K . Combination strategies with PD-1/PD-L1 blockade: current advances and future directions. Mol Cancer. (2022) 21:28. doi: 10.1186/s12943-021-01489-2, PMID: 35062949 PMC8780712

[22] Zamani MR Aslani S Salmaninejad A Javan MR Rezaei N . PD-1/PD-L and autoimmunity: A growing relationship. Cell Immunol. (2016) 310:27–41. doi: 10.1016/j.cellimm.2016.09.009, PMID: 27660198

[23] Raptopoulou AP Bertsias G Makrygiannakis D Verginis P Kritikos I Tzardi M . The programmed death 1/programmed death ligand 1 inhibitory pathway is up-regulated in rheumatoid synovium and regulates peripheral T cell responses in human and murine arthritis. Arthritis Rheum. (2010) 62:1870–80. doi: 10.1002/art.27500, PMID: 20506224

[24] Matsuda K Miyoshi H Hiraoka K Hamada T Yoshida S Ishibashi Y . Clinicopathological value of programmed cell death 1 (PD-1) and programmed cell death ligand 1 (PD-L1) expression in synovium of patients with rheumatoid arthritis. Clin Exp Med. (2018) 18:487–94. doi: 10.1007/s10238-018-0515-4, PMID: 29961175

[25] Liu C Jiang J Gao L Wang X Hu X Wu M . Soluble PD-1 aggravates progression of collagen-induced arthritis through Th1 and Th17 pathways. Arthritis Res Ther. (2015) 17:340. doi: 10.1186/s13075-015-0859-z, PMID: 26608464 PMC4659197

[26] Wang X Yang C Xu F Qi L Wang J Yang P . Imbalance of circulating Tfr/Tfh ratio in patients with rheumatoid arthritis. Clin Exp Med. (2019) 19:55–64. doi: 10.1007/s10238-018-0530-5, PMID: 30284646

[27] Bommarito D Hall C Taams LS Corrigall VM . Inflammatory cytokines compromise programmed cell death-1 (PD-1)-mediated T cell suppression in inflammatory arthritis through up-regulation of soluble PD-1. Clin Exp Immunol. (2017) 188:455–66. doi: 10.1111/cei.12949, PMID: 28245522 PMC5422858

[28] Nielsen C Ohm-Laursen L Barington T Husby S Lillevang ST . Alternative splice variants of the human PD-1 gene. Cell Immunol. (2005) 235:109–16. doi: 10.1016/j.cellimm.2005.07.007, PMID: 16171790

[29] de Almeida LGN Thode H Eslambolchi Y Chopra S Young D Gill S . Matrix metalloproteinases: from molecular mechanisms to physiology, pathophysiology, and pharmacology. Pharmacol Rev. (2022) 74:712–68. doi: 10.1124/pharmrev.121.000349, PMID: 35738680

[30] Cabral-Pacheco GA Garza-Veloz I Castruita-De la Rosa C Ramirez-Acuña JM Perez-Romero BA Guerrero-Rodriguez JF . The roles of matrix metalloproteinases and their inhibitors in human diseases. Int J Mol Sci. (2020) 21:9739. doi: 10.3390/ijms21249739, PMID: 33419373 PMC7767220

[31] Grillet B Pereira RVS Van Damme J Abu El-Asrar A Proost P Opdenakker G . Matrix metalloproteinases in arthritis: towards precision medicine. Nat Rev Rheumatol. (2023) 19:363–77. doi: 10.1038/s41584-023-00966-w, PMID: 37161083

[32] Bassiouni W Ali MAM Schulz R . Multifunctional intracellular matrix metalloproteinases: implications in disease. FEBS J. (2021) 288:7162–82. doi: 10.1111/febs.15701, PMID: 33405316

[33] Maybee DV Ink NL Ali MAM . Novel roles of MT1-MMP and MMP-2: beyond the extracellular milieu. Int J Mol Sci. (2022) 23:9513. doi: 10.3390/ijms23179513, PMID: 36076910 PMC9455801

[34] Eck SM Blackburn JS Schmucker AC Burrage PS Brinckerhoff CE . Matrix metalloproteinase and G protein coupled receptors: co-conspirators in the pathogenesis of autoimmune disease and cancer. J Autoimmun. (2009) 33:214–21. doi: 10.1016/j.jaut.2009.09.011, PMID: 19800199 PMC2783549

[35] Zhang Y Mi X Zhang Y Li J Qin Y He P . Immune checkpoint activity exacerbate renal interstitial fibrosis progression by enhancing PD-L1 expression in renal tubular epithelial cells. Transl Res. (2024) 271:52–67. doi: 10.1016/j.trsl.2024.05.004, PMID: 38723861

[36] Yang S Wang L Pan W Bayer W Thoens C Heim K . MMP2/MMP9-mediated CD100 shedding is crucial for inducing intrahepatic anti-HBV CD8 T cell responses and HBV clearance. J Hepatol. (2019) 71:685–98. doi: 10.1016/j.jhep.2019.05.013, PMID: 31173811

[37] Angelini G Flego D Vinci R Pedicino D Trotta F Ruggio A . Matrix metalloproteinase-9 might affect adaptive immunity in non-ST segment elevation acute coronary syndromes by increasing CD31 cleavage on CD4+ T-cells. Eur Heart J. (2018) 39:1089–97. doi: 10.1093/eurheartj/ehx684, PMID: 29211854 PMC5915953

[38] Li F Leier A Liu Q Wang Y Xiang D Akutsu T . Procleave: predicting protease-specific substrate cleavage sites by combining sequence and structural information. Genomics Proteomics Bioinf. (2020) 18:52–64. doi: 10.1016/j.gpb.2019.08.002, PMID: 32413515 PMC7393547

[39] Gamerith G Mildner F Merkel PA Harris K Cooney L Lim N . Association of baseline soluble immune checkpoints with the risk of relapse in PR3-ANCA vasculitis following induction of remission. Ann Rheum Dis. (2023) 82:253–61. doi: 10.1136/ard-2022-222479, PMID: 35973802 PMC12341842

[40] Bruzzaniti S Piemonte E Bruzzese D Lepore MT Strollo R Izzo L . Progression of type 1 diabetes is associated with high levels of soluble PD-1 in islet autoantibody-positive children. Diabetologia. (2024) 67:714–23. doi: 10.1007/s00125-023-06075-3, PMID: 38214712 PMC10904438

[41] Rauber S Mohammadian H Schmidkonz C Atzinger A Soare A Treutlein C . CD200+ fibroblasts form a pro-resolving mesenchymal network in arthritis. Nat Immunol. (2024) 25:682–92. doi: 10.1038/s41590-024-01774-4, PMID: 38396288

[42] Li Z Chen M Wang Z Fan Q Lin Z Tao X . Berberine inhibits RA-FLS cell proliferation and adhesion by regulating RAS/MAPK/FOXO/HIF-1 signal pathway in the treatment of rheumatoid arthritis. Bone Joint Res. (2023) 12:91–102. doi: 10.1302/2046-3758.122.BJR-2022-0269.R1, PMID: 36718649 PMC9950669

[43] Pitts SC Schlom J Donahue RN . Soluble immune checkpoints: implications for cancer prognosis and response to immune checkpoint therapy and conventional therapies. J Exp Clin Cancer Res. (2024) 43:155. doi: 10.1186/s13046-024-03074-z, PMID: 38822401 PMC11141022

[44] Sleeboom JJF van Tienderen GS Schenke-Layland K van der Laan LJW Khalil AA Verstegen MMA . The extracellular matrix as hallmark of cancer and metastasis: From biomechanics to therapeutic targets. Sci Transl Med. (2024) 16:eadg3840. doi: 10.1126/scitranslmed.adg3840, PMID: 38170791

[45] Mazor R Friedmann-Morvinski D Alsaigh T Kleifeld O Kistler EB Rousso-Noori L . Cleavage of the leptin receptor by matrix metalloproteinase-2 promotes leptin resistance and obesity in mice. Sci Transl Med. (2018) 10:eaah 6324. doi: 10.1126/scitranslmed.aah6324, PMID: 30135249 PMC9678493

[46] Choi E MaChado CR Okano T Boyle D Wang W Firestein GS . Joint-specific rheumatoid arthritis fibroblast-like synoviocyte regulation identified by integration of chromatin access and transcriptional activity. JCI Insight. (2024) 9:e179392. doi: 10.1172/jci.insight.179392, PMID: 38781031 PMC11383168

