# Supplementary figures and images for: Fibroblast-like synoviocytes mediate the generation of soluble PD-1 in an MMP-9-dependent manner: a novel target therapy for rheumatoid arthritis
Source: Front Immunol. 2025 Dec 10;16:1665078. doi: 10.3389/fimmu.2025.1665078 (PMC12727936; doi:10.3389/fimmu.2025.1665078)

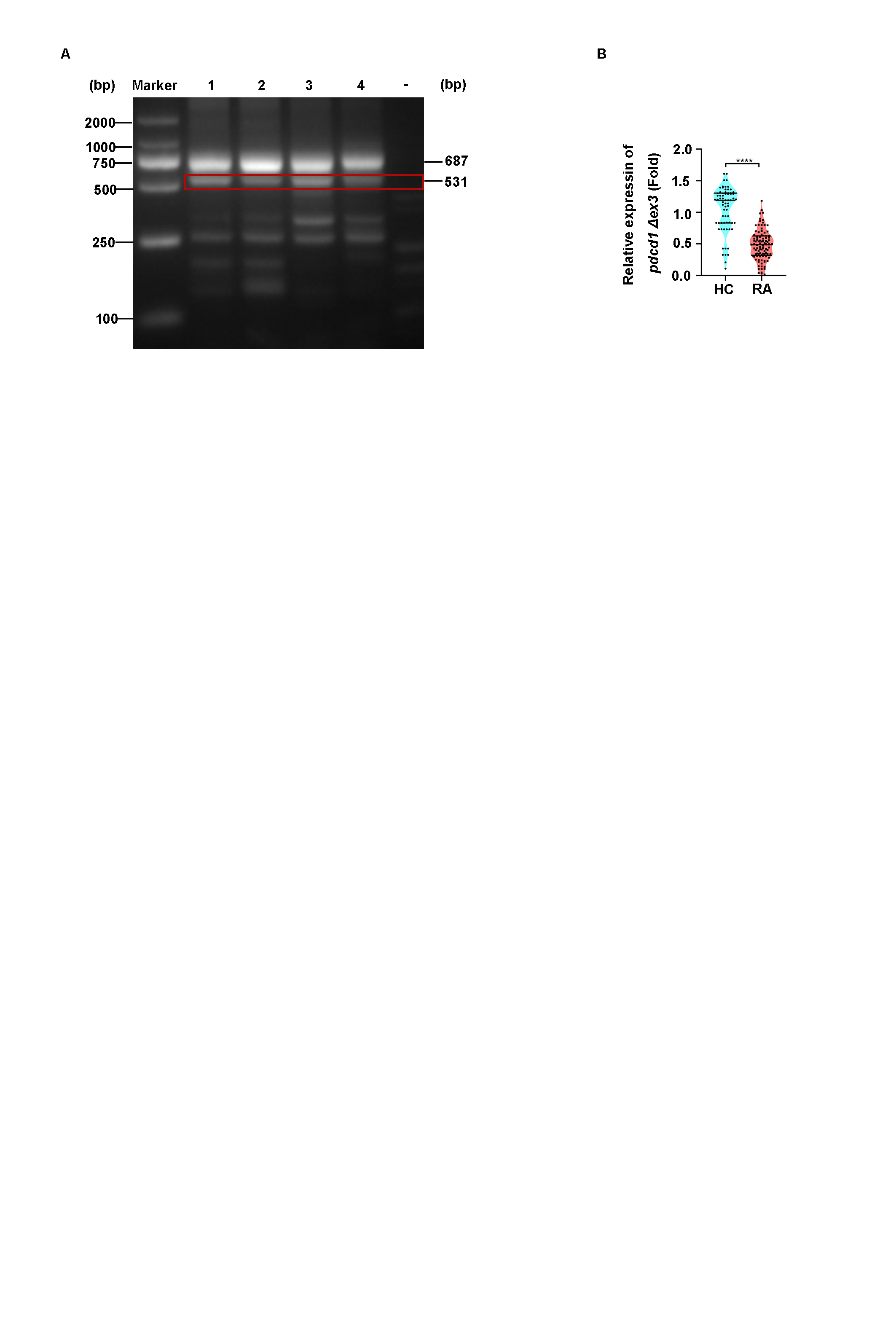

Supplement: Supplementary file 1 [file Image1.jpeg]

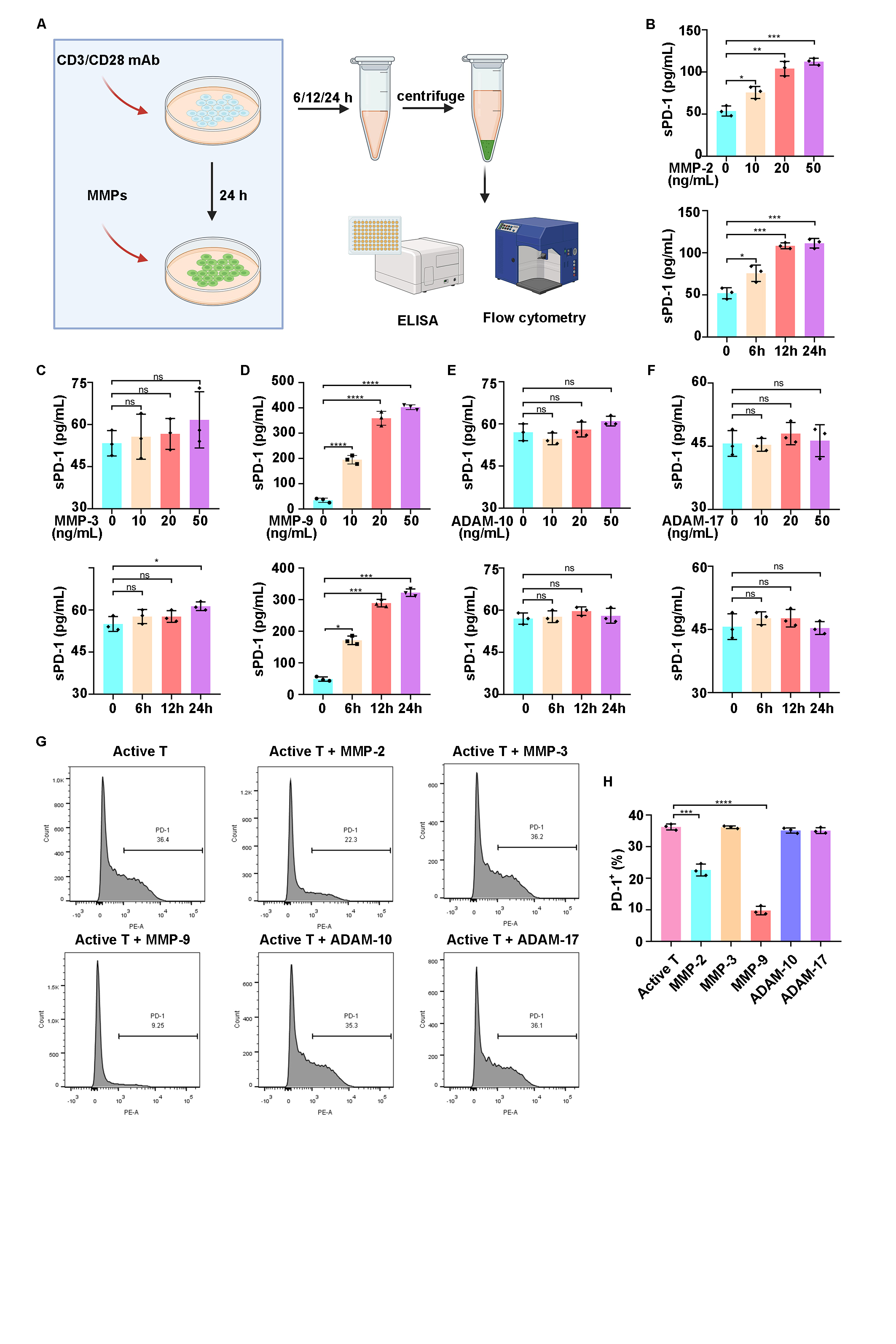

Supplement: Supplementary file 2 [file Image2.jpeg]

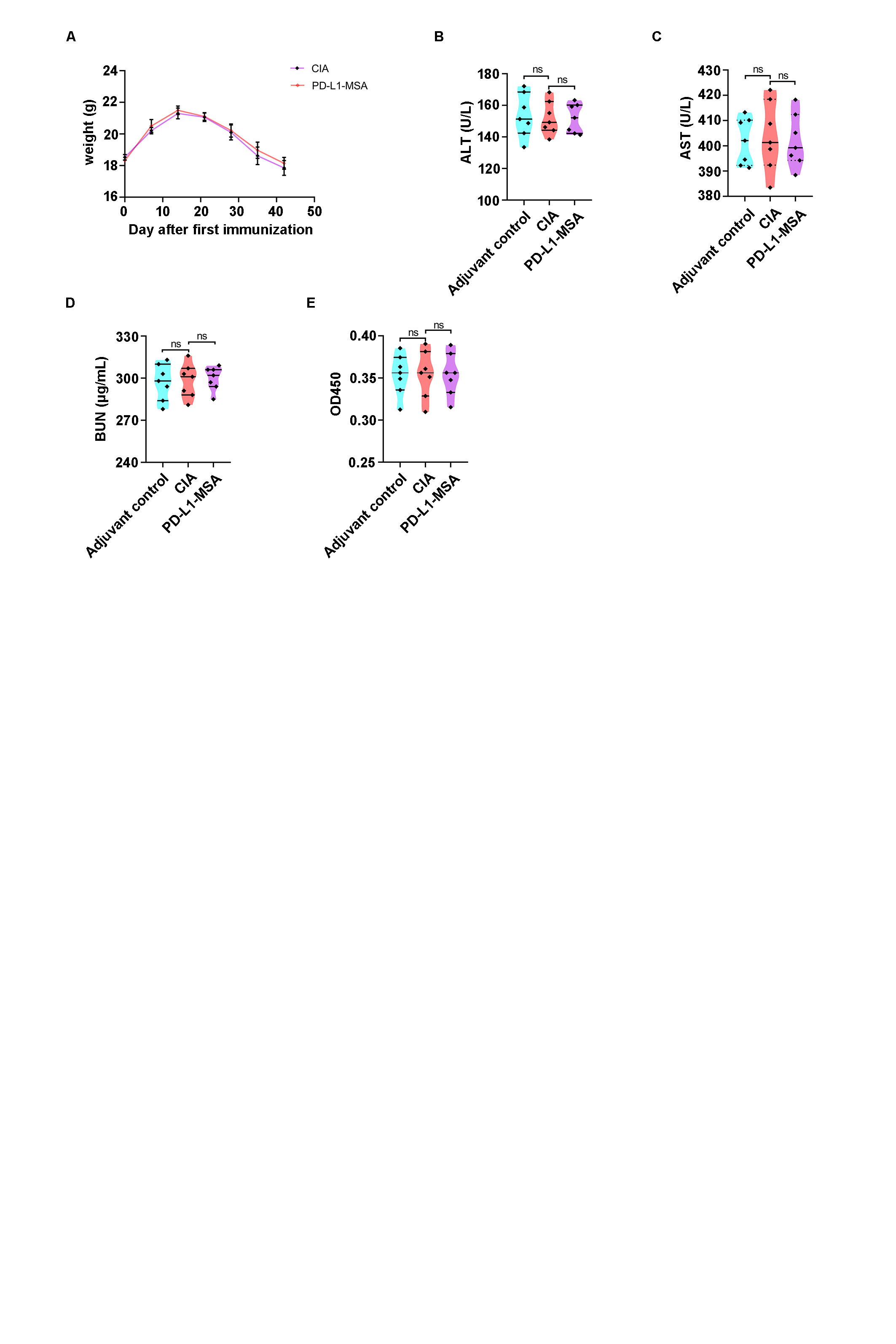

Supplement: Supplementary file 3 [file Image3.jpeg]

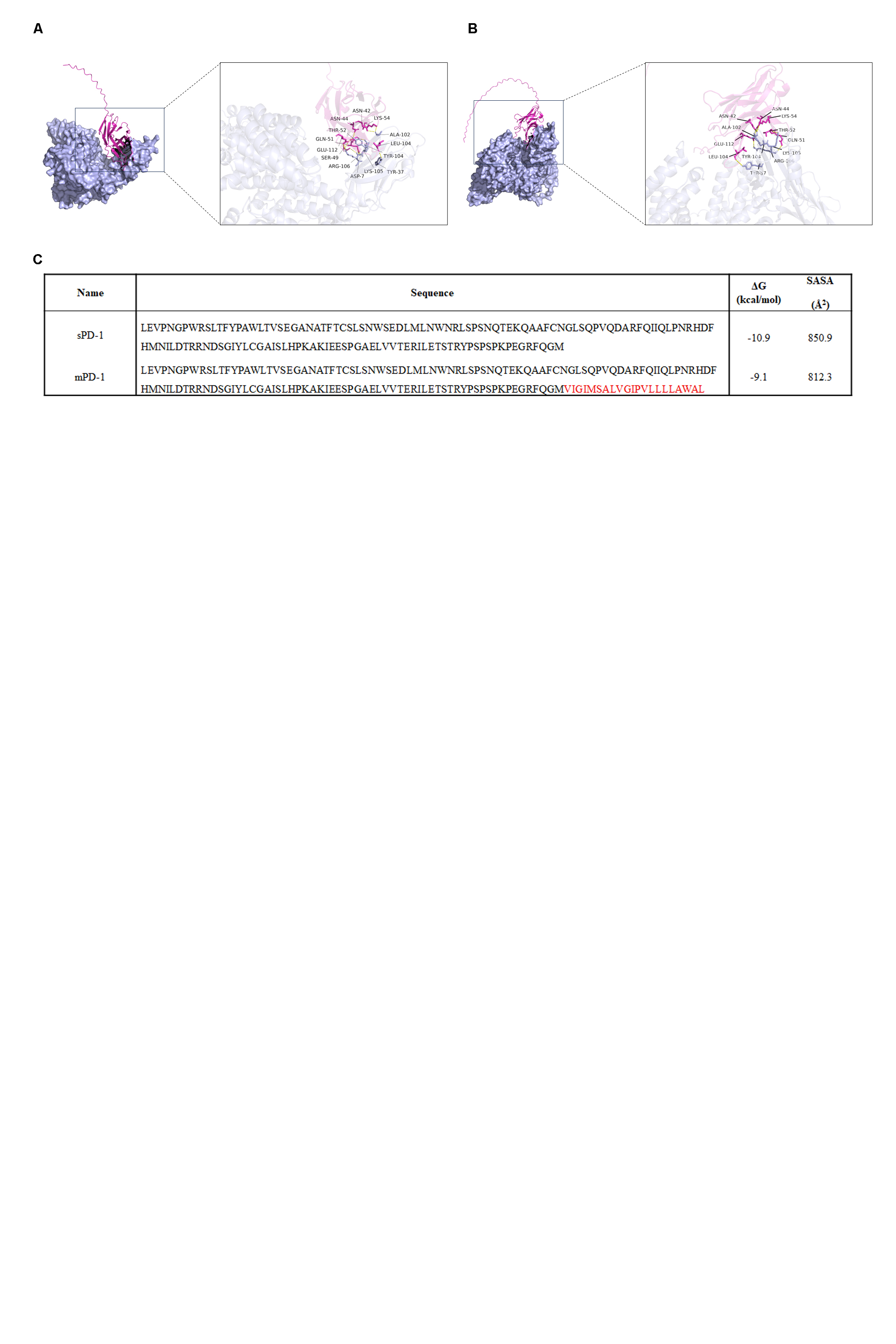

Supplement: Supplementary file 4 [file Image4.jpeg]
