# Supplementary material for: Fibroblast-like synoviocytes mediate the generation of soluble PD-1 in an MMP-9-dependent manner: a novel target therapy for rheumatoid arthritis
Source: Front Immunol. 2025 Dec 10;16:1665078. doi: 10.3389/fimmu.2025.1665078 (PMC12727936; doi:10.3389/fimmu.2025.1665078)
Supplement: Supplementary file 5 [file Table1.docx]

Supplementary Material

# Supplementary Figures and Tables

## Supplementary Figuress

**Supplementary Figure 1.** Detection of programmed cell death 1 protein lacking exon 3 (*pdcd1Δex3*) splicing variant in peripheral blood mononuclear cell (PBMC) of patients with RA. **(A)** Gel electrophoresis of PD-1 cDNA amplification products of PBMCs from patients with RA and healthy controls (HC). 1: HC; 2-4: RA; -: negative controls. **(B)** *pdcd1Δex3* expression were decreased in RA patients compared with HC individuals. Lane marker: DL2000 DNA Marker (Spark; Cat: AJ0101-A); Lane1: HC; Lane 2-4: RA; Lane -: negative controls; RA: Rheumatoid arthritis, HC: Health control, ****p <0.0001.

**Supplementary Figure 2.** Effects of various MMPs and ADAMs on PD-1 cleavage and sPD-1 secretion in Jurkat cells. **(A)** Schematic diagram of the experimental workflow: Jurkat cells were activated with CD3/CD28 monoclonal antibodies prior to stimulation with different proteases, followed by detection of sPD-1 levels in cell culture supernatants and PD-1 expression on the cell surface. **(B-F)** The levels of sPD-1 in the culture supernatant significantly increased in a time- and dose-dependent manner after administration of MMP-2 and MMP-9. **(G, H)** The flow cytometry results indicated a significant decrease in the PD-1 membrane surface signal of activated Jurkat cells after administration of MMP-2 and MMP-9. *p <0.05, **p <0.01, ***p <0.001, ****p <0.0001, ns, not significant.

**Supplementary Figure 3.** Safety and immunogenicity of PD-L1-MSA in a collagen-induced arthritis (CIA) mouse model **(A)** Dynamic monitoring of body weight in mice from the CIA group and PD-L1-MSA-treated group throughout the experimental period, no statistically significant differences were observed in body weight between the PD-L1-MSA-treated group and the CIA group. **(B-D)** Comparison of serum biochemical indicators between the two groups, including alanine aminotransferase (ALT), aspartate aminotransferase (AST), and blood urea nitrogen (BUN), No statistically significant differences were observed in serum ALT, AST, and BUN levels between the PD-L1-MSA-treated group and the CIA group. **(E)** No statistically significant differences were observed in the serum anti-PD-L1-MSA antibodies levels between the PD-L1-MSA-treated group and the CIA group. ns, not significant.

**Supplementary Figure 4.** AF2-predicted interface contacts of PD-L1-MSA/sPD-1 and PD-L1-MSA/mPD-1 complexes and analysis of binding free energy. **(A)** AF2 models depicting predicted interfaces of PD-L1-MSA/sPD-1 and PD-L1-MSA/mPD-1 complexes. **(B)** The binding free energy of PD-L1-MSA/sPD-1 and PD-L1-MSA/mPD-1 complexes were calculated using the Prodigy web-based tool, and PD-L1-MSA showed a higher binding affinity for sPD-1 compared with mPD-1.

## Supplementary Tables

**Supplementary Table 1** Previous medication history of the enrolled patients with RA.

| **Agent Type** | **Number of patients** | **Past Medication Dosages** | **Washout Period** | **Current Status** |
| --- | --- | --- | --- | --- |
| **No agent** | 72 | - | - | - |
| **csDMARDs** | 18 | MTX: 10-15 mg/week, n=7  LEF: 20 mg/day, n=5  IGU: 25 mg/day, n=6 | 6-9 months | discontinued |
| **bDMARDs** | 6 | Adalimumab: 40 mg/2 weeks, n=4  Tocilizumab: 8 mg/kg/4 weeks, n=2 | 8-12 months | discontinued |
| **GC** | 4 | Prednisone: 5-10 mg/day (4-8 weeks) | 7-10 months | discontinued |

**Supplementary Table 2** List of primer sequences used for q-PCR.

| **Gene** | **Forward primer** | **Reverse primer** |
| --- | --- | --- |
| *pdcd1 Δex3* | AGGGTGACAGGGACAATAGG | CCATAGTCCACAGAGAACAC |
| *mmp1* | ATGAAGCAGCCCAGATGTGGAG | TGGTCCACATCTGCTCTTGGCA |
| *mmp2* | GATACCCCTTTGACGGTAAGGA | CCTTCTCCCAAGGTCCATAGC |
| *mmp3* | GGAGATGCTCACTTCGATGAT | CAGCAACCAGGAATAAATTGG |
| *mmp9* | CCTCGCCCTGAACCTGAGC | GCTCTGAGGGGTGGACAGTG |
| *mmp12* | TGCTGATGACATACGTGGCA | AGGATTTGGCAAGCGTTGG |
| *mmp13* | AAATTATGGAGGAGATGCCCATT | TCCTTGGAGTGGTCAAGACCTAA |
| *mmp14* | GCCCAATGGGAAGACCTACT | AGGGTACTCGCTGTCCACTG |
| *gapdh* | AACTTTGGCATTGTGGAAGG | ACACATTGGGGGTAGGAACA |
